# BFMambaNet: Boundary-Frequency-Guided Global Semantic Mamba Network for Fine-Grained *Camellia oleifera* Leaf Disease Segmentation

**DOI:** 10.3390/plants15162493

**Published:** 2026-08-17

**Authors:** Xuanhao Li, Fulin Su, Yongming Yan, Shaofeng Peng, Lin Li, Fangying Wan, Ruifeng Liu

**Affiliations:** 1 School of Electronic Information and Physics, Central South University of Forestry and Technology, Changsha 410004, China; hao20233494@163.com (X.L.);; 2 Yuelushan Laboratory, Changsha 410128, China; 3 Hunan Academy of Forestry, Changsha 410004, China; 4 College of Forestry, Central South University of Forestry and Technology, Changsha 410004, China

**Keywords:** deep learning, *Camellia oleifera*, disease segmentation, Mamba, boundary-frequency guidance

## Abstract

*Camellia oleifera* leaf disease segmentation under natural field conditions is important for precision plant protection but remains challenging because lesions often show small target areas, blurred boundaries, uneven illumination, complex backgrounds, and coexisting symptoms. To address these problems, this paper proposes BFMambaNet, a Boundary-Frequency-guided Global Semantic Mamba Network for fine-grained disease segmentation. The model adopts an encoder-decoder framework and introduces a Global Semantic Mamba-based spatial selective feature modeling block to capture long-range lesion context and reduce semantic confusion. A gated wavelet spatial enhancement block is further designed to strengthen high-frequency boundary details while suppressing noisy responses. During training, boundary-frequency auxiliary supervision guides contour localization and pathological texture recovery without additional manual boundary labels. A reinforcement-learning-guided adaptive loss controller adjusts class-wise reweighting factors and loss-component weights according to the training state, improving optimization stability. A pixel-level dataset containing 1400 images and seven disease categories was constructed for evaluation. Experimental results show that BFMambaNet achieves 92.39% Precision, 91.43% Recall, 91.26% Dice, and 85.46% mIoU, outperforming representative CNN-based, Transformer-based, and Mamba-based models. Evaluations on environmental subsets confirm superior robustness, outperforming VMamba by 3.70% mIoU under uneven illumination, 3.55% mIoU under complex backgrounds, and 5.10% mIoU under coexisting symptoms. Cross-dataset validation on Apple leaf diseases further proves its generalization with 3.39% mIoU and 3.84% Dice improvements over U-Mamba, while maintaining a competitive inference speed of 30 FPS. Qualitative results also show clearer boundaries, fewer missed small lesions, and more stable predictions in complex field scenarios.

## 1. Introduction

*Camellia oleifera* is an important woody oil crop with high economic and nutritional value. Its seeds are widely used for producing camellia oil, and related studies have reported the value of *Camellia oleifera* and its by-products in food, medicine, and industrial applications [1]. The fatty-acid composition of *Camellia oleifera* oil is rich in unsaturated fatty acids, especially oleic acid, which further supports its value as a high-quality edible oil resource. In addition, bioactive components from *Camellia oleifera* have shown anti-inflammatory and antioxidative potential [2,3,4]. Therefore, stable production and healthy cultivation of *Camellia oleifera* are important for both agricultural economy and downstream industrial utilization.

However, *Camellia oleifera* is frequently affected by leaf and fruit diseases in natural cultivation environments. Among them, anthracnose is one of the major threats to the sustainable development of the *Camellia oleifera* industry. Previous studies have shown that pathogens such as *Colletotrichum* species can cause severe lesions and reduce plant health, and biological-control strategies have been explored to suppress disease development [5,6]. More broadly, plant diseases remain a serious threat to global food security because they reduce yield, quality, and production stability [7]. Early and accurate disease monitoring is therefore essential for disease control, precise management, and yield protection.

Traditional plant disease monitoring still relies heavily on manual field inspection. This process is labor-intensive, subjective, and difficult to scale to large plantations. Reviews of plant disease detection have emphasized that rapid, reliable, and non-destructive imaging technologies are needed for modern agricultural monitoring [8,9]. Early automatic methods often used color thresholds, texture descriptors, morphological operations, and soft-computing algorithms to segment diseased regions [10]. These methods can work under controlled conditions, but they are sensitive to illumination changes, lesion color variation, background interference, and parameter selection. Similar conclusions have also been observed in broader image-analysis studies, where traditional image processing methods usually require handcrafted features and show limited adaptability compared with learning-based approaches [11,12,13].

With the development of machine learning and deep learning, image-based plant disease detection has achieved significant progress. Deep convolutional neural networks can automatically learn discriminative features from plant leaf images and have demonstrated strong performance in disease classification tasks [14]. Compared with image-level classification, semantic segmentation provides pixel-level lesion localization, which is more useful for estimating disease severity and analyzing lesion morphology. For example, deep learning-based segmentation methods have been used to extract disease-affected regions from leaf images and support subsequent disease classification [15]. These studies show that segmentation is a key step for fine-grained plant disease analysis.

Convolutional neural networks have become a major foundation for dense prediction tasks. CNN-based segmentation models have been widely applied in medical and natural image segmentation because they can learn local texture and spatial patterns from data [16]. Some CNN-based methods further encode semantic segmentation-aware features to improve localization sensitivity [17]. Spatiotemporal CNNs also show that multi-scale feature aggregation and attention-like refinement can improve segmentation quality in complex scenes [18]. Beyond agricultural computer vision, neural network-enhanced diagnostics have demonstrated robust performance in capturing subtle structural anomalies, such as internal leakage detection in hydraulic actuators [19], showcasing the cross-domain utility of deep representations in structural diagnostics. These works demonstrate the effectiveness of CNNs in extracting local visual patterns. However, convolutional operations are inherently limited by local receptive fields. For plant disease images with scattered lesions and confusing background textures, local features alone may not be sufficient for robust global discrimination.

To improve contextual representation, several semantic segmentation architectures have introduced multi-scale context modeling. DeepLab uses atrous convolution and atrous spatial pyramid pooling to enlarge the receptive field and capture objects at multiple scales [20]. PSPNet-style segmentation methods further show the importance of spatial context aggregation for dense prediction [21]. High-resolution networks preserve detailed spatial representations during feature extraction, which is valuable for tasks requiring accurate localization [22]. These models improve segmentation performance by enhancing scale awareness and spatial detail preservation. Nevertheless, plant disease segmentation remains challenging because small lesions may occupy only a limited number of pixels, and their boundaries are often weak, irregular, and low contrast.

Transformer-based models provide another direction for global context modeling. The self-attention mechanism introduced by Transformer can capture long-range dependencies and has influenced many vision architectures [23]. Vision Transformer variants further adapt attention to image patches and improve representation learning for visual tasks [24]. However, attention-based global modeling usually brings higher computational cost, especially for high-resolution images. In agricultural disease segmentation, images often contain fine lesion details and complex backgrounds. This creates a need for models that can capture global context while maintaining computational efficiency.

Recently, Mamba and state-space models have provided a promising alternative for efficient long-range modeling. VMamba adapts Mamba to visual feature modeling through visual state-space blocks and selective scanning, allowing global dependency modeling with linear complexity [25]. A broader survey of Mamba-based models also highlights the potential of selective state-space models as efficient alternatives to attention-based architectures [26]. In remote sensing, Vision Mamba has been discussed as a promising solution for high-resolution visual understanding because CNNs are constrained by limited receptive fields and ViTs by quadratic complexity [27]. These properties are also meaningful for plant disease segmentation. Lesions may appear in scattered regions across the leaf surface, and global semantic relationships can help distinguish true disease regions from leaf veins, shadows, and background noise.

Despite these advances, global semantic modeling alone cannot fully solve the segmentation problem. Disease boundaries are often fuzzy and fragmented, while small lesions may lose high-frequency details during repeated downsampling and upsampling. Wavelet-domain analysis provides a useful way to represent edges, ridges, and texture changes at multiple scales. Early wavelet-based segmentation studies showed that wavelet coefficients are well suited for capturing singularities and texture structures [28]. More recent wavelet-attention segmentation methods also demonstrate that wavelet features can enhance structural details in dense prediction tasks [29]. Therefore, incorporating wavelet-guided high-frequency information is a reasonable strategy for improving lesion boundary perception and preserving fine pathological textures.

Another key challenge is optimization imbalance. In plant disease segmentation, background and healthy leaf pixels often dominate the image, while lesion pixels are much fewer. This imbalance weakens the gradient contribution of small or difficult lesion regions. At the same time, different training stages may require different optimization emphases. Early training needs stable region learning, whereas later training may benefit more from boundary and frequency refinement. Reinforcement learning has been explored for image segmentation because it can model the segmentation process as a sequential decision-making problem and progressively improve segmentation quality [30]. Multi-agent reinforcement learning has also been used to enhance medical image segmentation, showing the potential of adaptive decision strategies in segmentation tasks [31]. These studies motivate the use of a reinforcement-learning-guided controller to adaptively adjust loss weights during training.

Boundary modeling and intermediate supervision are also important for dense prediction. Deeply supervised learning provides direct optimization signals to intermediate layers, which can improve gradient flow and strengthen multi-scale representation learning [32,33]. Meanwhile, common segmentation losses, such as cross-entropy and Dice loss, mainly measure region-level agreement. They may not sufficiently penalize contour displacement when foreground regions are small or highly imbalanced. Boundary loss addresses this limitation by introducing contour-based constraints for highly unbalanced segmentation [34]. Boundary-aware loss terms can further complement cross-entropy by explicitly modeling the transformation between predicted and target boundaries [35]. These studies suggest that explicit boundary constraints and auxiliary supervision are useful for improving structural integrity and training stability.

**Figure 1:**
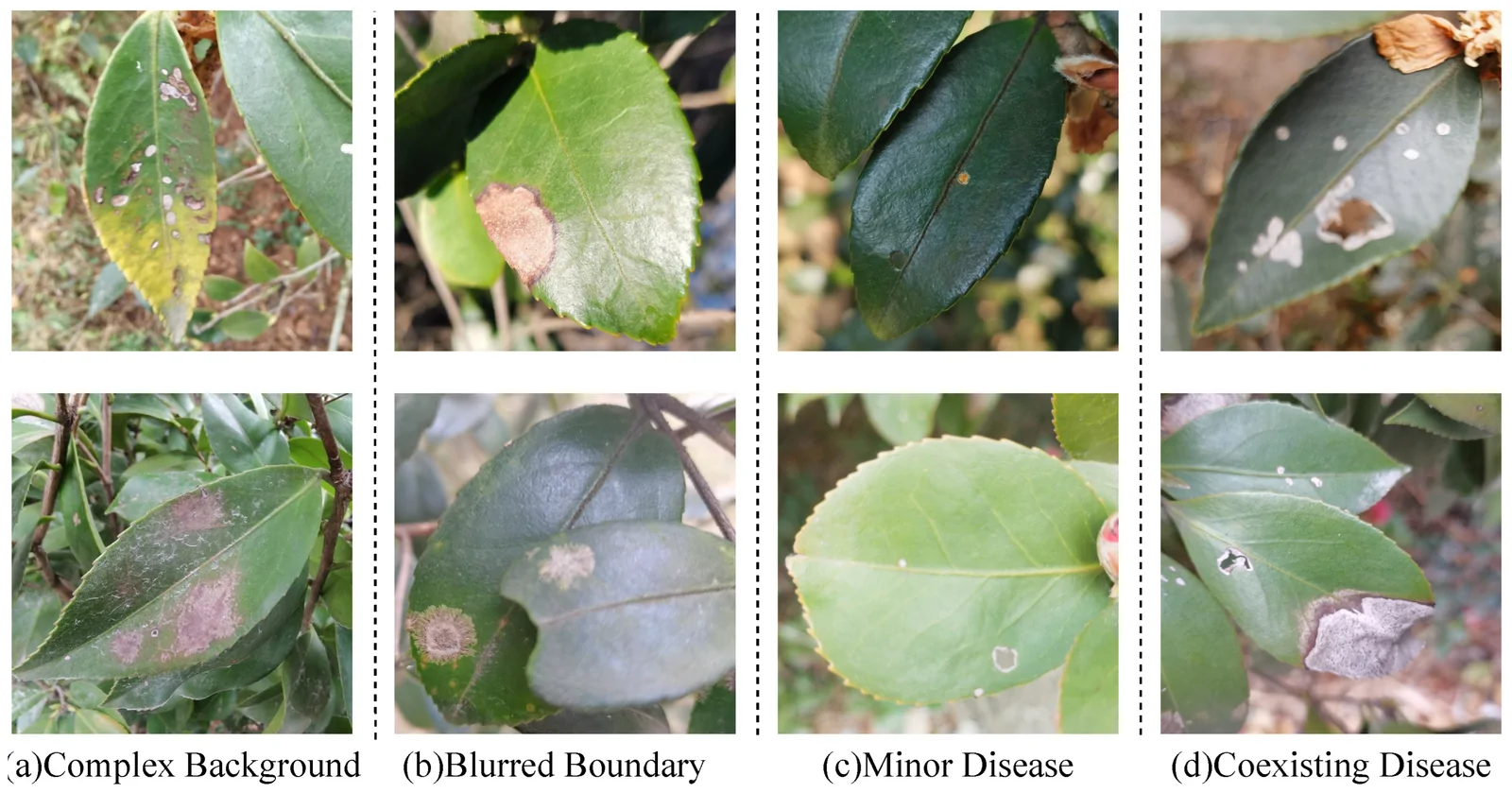
Representative challenges in fine-grained *Camellia oleifera* leaf disease segmentation. (**a**) Complex background, (**b**) blurred boundary, (**c**) minor disease, and (**d**) coexisting disease.

Representative challenging cases are shown in Figure 1. These examples illustrate four common difficulties in field-acquired *Camellia oleifera* disease images: complex backgrounds, blurred lesion boundaries, minor disease regions, and coexisting disease symptoms.

In summary, despite the progress of deep learning in plant disease segmentation, several challenges still persist:

1. **Lack of a Unified Disease Segmentation Framework:** Existing methods often address semantic modeling, boundary refinement, auxiliary supervision, and loss optimization as separate problems. This limits their ability to jointly handle scattered lesions, weak contours, and unstable training in complex field images.
2. **Insufficient Long-Range Semantic Modeling:** Local convolutional features are effective for texture extraction, but they are not sufficient to capture spatial dependencies among scattered lesion regions. As a result, visually similar leaf veins, shadows, and background textures may be confused with true disease areas.
3. **Weak Boundary and Fine-Detail Degradation:** Although context-aware networks improve multi-scale representation, small pathological structures are still easily blurred or lost during repeated downsampling and upsampling. This often leads to incomplete lesion masks and inaccurate boundary localization.
4. **Limited Structural Supervision:** Region-based losses mainly measure pixel-level overlap and cannot fully constrain contour displacement. In addition, deep networks without sufficient intermediate supervision may suffer from unstable gradient propagation and weak multi-scale feature learning.
5. **Imbalanced and Stage-Dependent Optimization:** Fixed loss weights cannot fully adapt to class imbalance and changing training requirements. Early training requires stable region learning, whereas later training needs stronger boundary and frequency refinement.

To address these challenges, we propose BFMambaNet, a Boundary-Frequency-guided Global Semantic Mamba Network for fine-grained plant disease segmentation. The proposed framework integrates a Global Semantic Mamba-based spatial selective feature modeling module to capture long-range lesion context. It further introduces a gated wavelet spatial enhancement module to strengthen boundary-sensitive high-frequency responses. In addition, boundary-frequency auxiliary supervision is designed to provide explicit structural constraints during training. Finally, a reinforcement-learning-guided adaptive loss controller dynamically adjusts both class-wise reweighting factors and the weights of segmentation, boundary, and frequency losses according to the training state. Through these designs, BFMambaNet aims to improve lesion localization, boundary integrity, and optimization stability under complex field conditions.

The main contributions of this study are summarized as follows:

1. We propose BFMambaNet, an encoder-decoder segmentation framework that integrates global semantic modeling, wavelet-guided enhancement, boundary-frequency auxiliary supervision, and adaptive loss control for fine-grained plant disease segmentation.
2. We design a Global Semantic Mamba-based SSFM block to capture spatial dependencies among scattered lesions and improve discrimination between true lesions and visually similar background textures.
3. We introduce a Gated Wavelet Spatial Enhancement block that exploits high-frequency wavelet responses for weak boundary enhancement and fine lesion detail preservation.
4. We construct an integrated boundary-frequency auxiliary supervision strategy. Boundary targets are automatically generated from ground-truth masks through morphological operations and are used to guide both contour and frequency-detail learning.
5. We develop an RL-guided adaptive loss controller that outputs class-wise and component-wise actions. It adjusts class reweighting factors and the component weights of segmentation, boundary, and frequency losses, reducing optimization bias caused by fixed loss settings and improving training stability.

## 2. Related Work

### 2.1. Deep Learning-Based Segmentation in Agricultural Images

Deep learning has become the dominant paradigm for visual analysis in agriculture because it can learn discriminative features directly from images instead of relying on manually designed color or texture descriptors. CNN-based methods have been widely reviewed for plant leaf disease recognition, and their success mainly comes from hierarchical feature extraction, local pattern learning, and end-to-end optimization [36]. Although many early studies focused on image-level classification, pixel-level segmentation is more informative for disease severity estimation because it provides lesion location, area, and shape. In agricultural phenotyping, the comparison between Mask R-CNN and thresholding-based segmentation also shows that learning-based segmentation is more robust than rule-based thresholding when image conditions are not perfectly controlled [37]. This observation is relevant to plant disease segmentation, where leaf color, illumination, and background texture vary strongly in field images. To handle complex field and orchard conditions, specialized segmentation architectures such as EDSC-HRAFNet [38] have been proposed for extracting multi-level topological features of tree branches under challenging backgrounds. Similarly, in crop and seedling monitoring under flooded paddy conditions, unsupervised geometry-driven methods incorporating digital surface model (DSM) terrain correction [39] have been developed to separate target structures from specular reflections and terrain trends without manual annotations. Beyond color-channel transformations, multi-modal RGB-D data augmentation techniques [40] have also proven effective in boosting model generalization across diverse agronomic scenes.

Classical CNN segmentation networks provide useful design principles for dense prediction. DeepLab enlarges the receptive field through atrous convolution and contextual aggregation [20], PSPNet emphasizes pyramid-level spatial context [21], and high-resolution networks preserve detailed spatial representations during feature extraction [22]. These methods are effective for general semantic segmentation and have inspired many agricultural segmentation pipelines. However, they still have two limitations when applied to fine-grained lesion segmentation. First, convolutional features are mainly local, so scattered lesions may be interpreted independently rather than as semantically related pathological regions. Second, small disease regions and weak contours can be suppressed during repeated downsampling. Therefore, CNN-based segmentation provides a strong foundation, but it is not sufficient to fully handle small lesions, fuzzy boundaries, and visually similar leaf textures.

### 2.2. Transformer and Foundation Segmentation Models

Transformer-based segmentation methods address the locality limitation of CNNs by using attention to model long-range dependencies. SegFormer provides a simple and efficient segmentation framework by combining a hierarchical Transformer encoder with a lightweight MLP decoder [41]. Its design demonstrates that multi-scale Transformer features can improve dense prediction without relying on heavy decoders. Mask2Former further shows the strength of query-based masked attention and extends a unified segmentation formulation to video instance segmentation [42]. More recently, promptable foundation models such as SAM 2 have shown strong general segmentation ability across images and videos through large-scale training and interactive prompts [43]. Building upon promptable segment-anything architectures, X-SAM [44] further extends SAM-based paradigms from prompt-driven segment anything to universal segmentation across diverse visual tasks. These studies indicate that modern segmentation is moving toward stronger global modeling, more flexible decoding, and broader task generalization.

Despite their strong representation ability, Transformer and foundation segmentation models are not always ideal for plant disease segmentation. Attention-based methods usually require considerable computation for high-resolution inputs. More importantly, their general-purpose representations may not focus sufficiently on subtle pathological structures. Plant lesions are often small, irregular, and low contrast. They may also be confused with shadows, veins, natural spots, or background fragments. Promptable models can provide flexible segmentation priors, but they are not designed as fully automatic disease segmentation systems and may require prompts or large-scale adaptation. Therefore, a task-specific architecture is still needed. It should retain global context modeling while preserving fine local details and remaining efficient for high-resolution leaf images.

### 2.3. State Space Models and Mamba-Based Visual Modeling

State space models have recently attracted attention as efficient alternatives to attention for long-range sequence modeling. Mamba introduces selective state-space modeling and enables linear-time sequence processing by making the state transition input-dependent [45]. This property allows the model to selectively propagate informative tokens while filtering irrelevant information. For visual tasks, VMamba adapts selective scanning to image features and provides an efficient way to model global dependencies in two-dimensional feature maps [25]. These characteristics are useful for dense prediction because they offer a compromise between the local inductive bias of CNNs and the global modeling ability of Transformers.

Mamba-based architectures have also been explored in segmentation. Mamba-UNet builds a UNet-like pure visual Mamba architecture for medical image segmentation and shows the potential of Mamba blocks in encoder-decoder structures [46]. U-Mamba further combines convolutional feature extraction with state-space modeling to enhance long-range dependency in biomedical image segmentation [47]. These studies suggest that Mamba-based models can provide efficient global context while maintaining compatibility with dense prediction frameworks. However, most existing Mamba segmentation studies focus on medical or general visual domains. Their adaptation to plant disease segmentation remains less explored. Plant disease images contain irregular lesion distributions, heterogeneous leaf textures, and weak boundary transitions. These characteristics require not only long-range semantic scanning but also boundary-sensitive and frequency-aware refinement. This motivates the design of a Global Semantic Mamba-based spatial selective feature modeling module in BFMambaNet.

### 2.4. Boundary-Frequency Supervision and Adaptive Loss Optimization

Recent studies dedicated to *Camellia oleifera* disease segmentation have also shown the importance of combining visual features with semantic priors and adaptive optimization cues. LUNet integrates language information with UNet for pest and disease segmentation in complex agricultural environments, TB-DLossNet introduces semantic-visual fusion with boundary-aware and dynamic-loss designs, and RLEM-Net incorporates multimodal semantic-visual features with a reinforcement-learning weight adjuster for disease segmentation [48,49,50].

Frequency-domain learning provides another way to preserve structural details. Focal frequency loss shows that frequency-domain discrepancies can complement spatial losses and help models focus on hard frequency components [51]. Wavelet-based image segmentation also demonstrates that high-frequency components are useful for representing edges, ridges, and texture discontinuities [28,29]. For plant lesions, these high-frequency cues are closely related to weak boundaries and fine pathological textures. However, directly enhancing all high-frequency responses may also amplify background noise. Therefore, boundary and frequency information should be introduced selectively and under proper supervision. In BFMambaNet, boundary targets are generated from ground-truth masks, and boundary-frequency auxiliary heads are used only during training to guide contour localization and high-frequency detail recovery.

Accurate lesion segmentation depends not only on regional classification but also on structural detail preservation. Boundary-aware learning has been shown to improve contour alignment in semantic segmentation. Active boundary loss explicitly encourages predicted boundaries to move toward ground-truth boundaries and improves boundary quality beyond standard cross-entropy supervision [52]. Boundary loss is also effective for highly unbalanced segmentation because it constrains shape and contour errors instead of relying only on region overlap [34]. In addition, deeply supervised learning provides intermediate optimization signals and can improve gradient propagation in deep networks [32,33]. These methods indicate that auxiliary supervision is valuable when the final segmentation loss alone cannot fully constrain small or weak structures.

Loss weighting is another critical issue in plant disease segmentation. Fixed loss coefficients may be suboptimal because the training objective changes over time. Early epochs require stable region learning, whereas later epochs often require stronger structural refinement. Reinforcement learning provides a mechanism for adaptive decision-making based on training feedback. Deep reinforcement learning has been used for image segmentation in quantitative analysis tasks [53], while multi-step and multi-agent reinforcement learning strategies have also been investigated for medical image segmentation [30,31]. Recent studies further show that segmentation and reinforcement learning can benefit each other. SAM-R1 introduces SAM-based reward feedback for reinforcement learning in multimodal segmentation [54], and Focus-Then-Decide uses segmentation-assisted reinforcement learning to focus decision-making on task-relevant regions [55]. These works support the idea that reinforcement learning can guide segmentation-related optimization beyond fixed handcrafted rules.

Nevertheless, existing adaptive strategies usually focus on sample selection, task reasoning, or general decision policies. They rarely connect training-state feedback with the joint weighting of segmentation, boundary, and frequency objectives in plant disease segmentation. BFMambaNet addresses this gap by introducing an actor–critic adaptive loss controller. The controller observes class-wise Dice performance, validation-loss trend, and epoch progress. It then outputs both class-wise raw actions and component-wise raw actions, which are transformed into class reweighting factors and loss-component weights. In this way, the model can adapt its optimization emphasis according to the current training state while maintaining explicit supervision for region consistency, boundary accuracy, and frequency-detail recovery.

## 3. Materials and Methods

### 3.1. Data Collection

**Figure 2:**
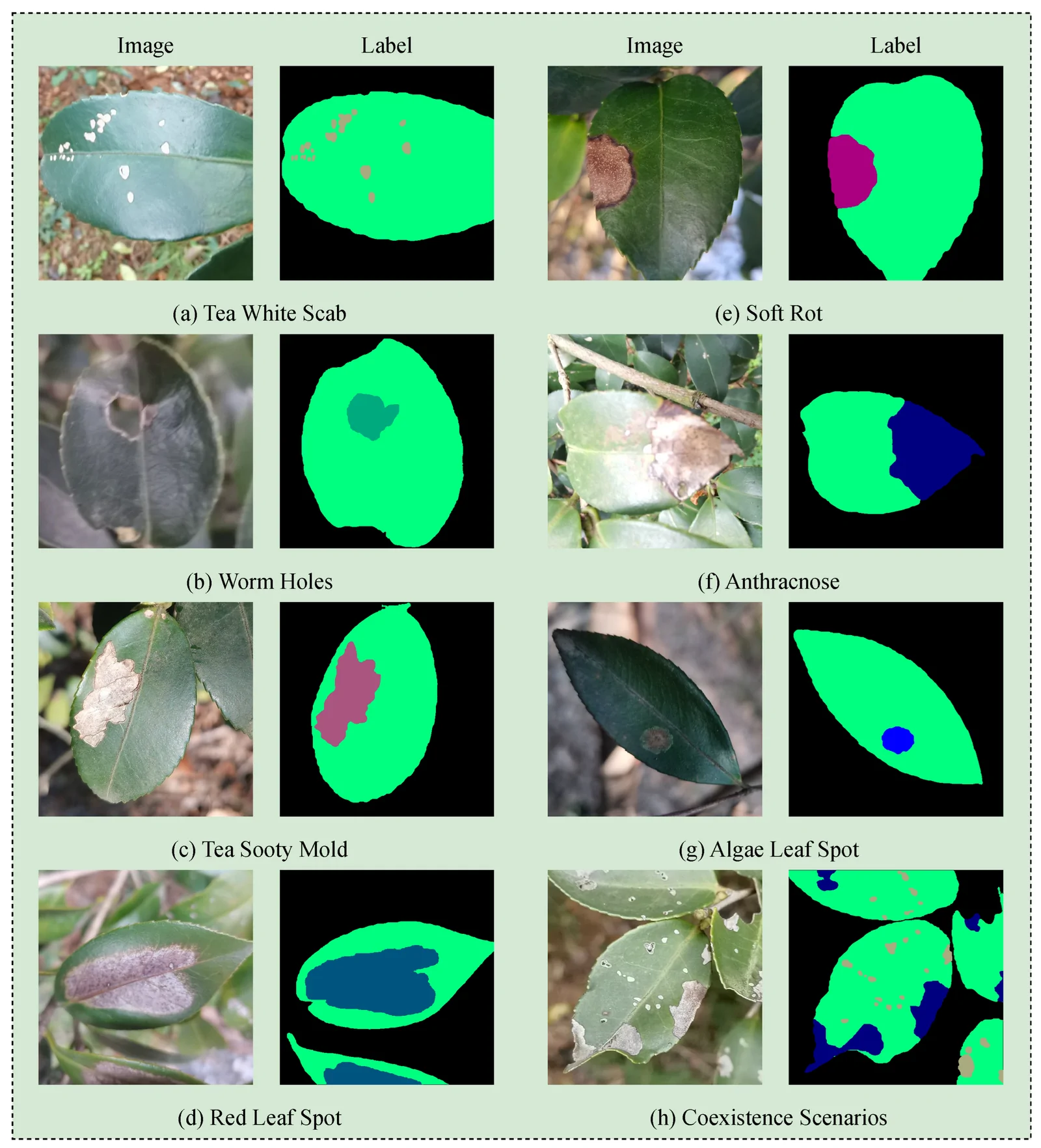
Representative examples of the *Camellia oleifera* leaf disease segmentation dataset. Each disease category is shown with its original RGB image and corresponding pixel-level label. The dataset includes seven common disease categories and coexistence scenarios with multiple lesion types.

The dataset used in this study was constructed for pixel-level segmentation of *Camellia oleifera* leaf diseases under complex field conditions. It contains RGB leaf images and corresponding semantic segmentation masks, which were used to train and evaluate the proposed BFMambaNet. Representative examples of the image–label pairs are shown in Figure 2.

Images were collected from *Camellia oleifera* plantations in three distinct geographic regions (Changde, Changsha, and Linli in Hunan Province, China) spanning multiple growth cycles from April 2023 to June 2025. All samples were captured in natural field environments using a mobile imaging device (Apple iPhone 14), utilizing the native wide-angle camera sensor with default automatic shooting parameters (auto-exposure, auto-focus, and JPEG compression). The original images were acquired at a native resolution of 3456 × 3456 pixels, maintaining a working distance of 5–10 cm under varied illumination, shooting angles, and background clutter. To balance computational efficiency and the preservation of lesion boundaries, all images were resized to 512 × 512 pixels prior to model training.

To rigorously evaluate the model’s performance and prevent potential data leakage—a critical issue in agricultural datasets where randomly splitting consecutively captured frames of the same leaf can lead to severe performance overestimation—the dataset was partitioned using a 5-fold cross-validation scheme. Crucially, during data collection, strict protocols were enforced to avoid burst-mode imaging or highly repetitive captures of the identical lesion. Furthermore, the 5-fold partitioning was conducted such that images derived from the same physical plant or localized temporal cluster were kept strictly separated across training, validation, and testing boundaries. This separation ensures that our reported test metrics genuinely reflect the model’s generalization capabilities to unseen pathological instances, directly addressing the risks of spatial and temporal data leakage.

The dataset comprises 1400 images covering seven common *Camellia oleifera* leaf disease categories: Tea White Scab, Worm Holes, Tea Sooty Mold, Red Leaf Spot, Soft Rot, Anthracnose, and Algae Leaf Spot. In addition, some images contain coexistence scenarios where multiple disease symptoms appear simultaneously on the same leaf or within the same image. These cases increase the complexity of the segmentation task because different lesions may vary greatly in scale, color, texture, and boundary clarity. The number of images containing each disease category is summarized in Table 1. Since some images contain multiple disease types, the category counts are not mutually exclusive, and the proportions do not sum to 100%.

Pixel-level annotations were generated for both leaf regions and disease lesions. The labeling process was performed with the assistance of an interactive annotation platform integrated with the Segment Anything Model (SAM), followed by manual inspection and refinement to ensure annotation quality. During annotation, particular attention was paid to lesion boundaries, small disease spots, and irregular pathological regions, so that the resulting masks could provide reliable supervision for fine-grained segmentation. The annotation format follows a semantic segmentation setting, where each pixel is assigned to the background, leaf tissue, or a specific disease category.

The category distribution is imbalanced. Tea White Scab appears in the largest number of images, whereas Anthracnose appears in the fewest images. This imbalance reflects the natural occurrence frequency of different diseases in field environments and also poses additional challenges for robust multi-class segmentation.

For model development, the dataset was divided into training, validation, and testing subsets. In our nnUNet-based experimental setting, 1260 images were used for training and validation under a five-fold cross-validation scheme, and the remaining 140 images were reserved as a fixed, held-out independent test set that was never used during model training, hyperparameter selection, or fold assignment. For each fold, 1008 images were used for training and 252 images were used for validation. The five-fold partitioning followed the default stratified splitting strategy of the nnUNet framework, which preserves the approximate proportion of each disease category across all folds, thereby ensuring consistent class representation despite the inherent class imbalance in the dataset. All quantitative results reported in the main comparison and ablation tables (Tables 5–12) are presented as mean ± standard deviation computed across the five validation folds, directly reflecting cross-validation stability and model reliability. The fixed independent test set (140 images) is used exclusively for the robustness evaluation on challenging subsets (Section 4.9), providing an additional out-of-distribution generalization assessment that is fully decoupled from the cross-validation procedure.

Data augmentation was applied to the training set to improve robustness under heterogeneous field conditions. The augmentation operations included random rotation, flipping, scaling, cropping, brightness and contrast adjustment, and other spatial or photometric transformations. These strategies were used to reduce overfitting and enhance the model’s ability to generalize to variations in leaf appearance, lesion morphology, and environmental background.

### 3.2. Proposed Method

To improve fine-grained plant disease segmentation under complex field conditions, we propose BFMambaNet, a Boundary-Frequency-guided Global Semantic Mamba Network. The framework is designed for leaf images with small lesions, weak boundaries, uneven illumination, and background textures that are visually close to disease regions. Instead of relying only on local convolutional features, BFMambaNet integrates global semantic feature modeling, wavelet-guided spatial enhancement, boundary-frequency auxiliary supervision, and reinforcement-learning-based adaptive loss control into a unified segmentation framework.

**Figure 3:**
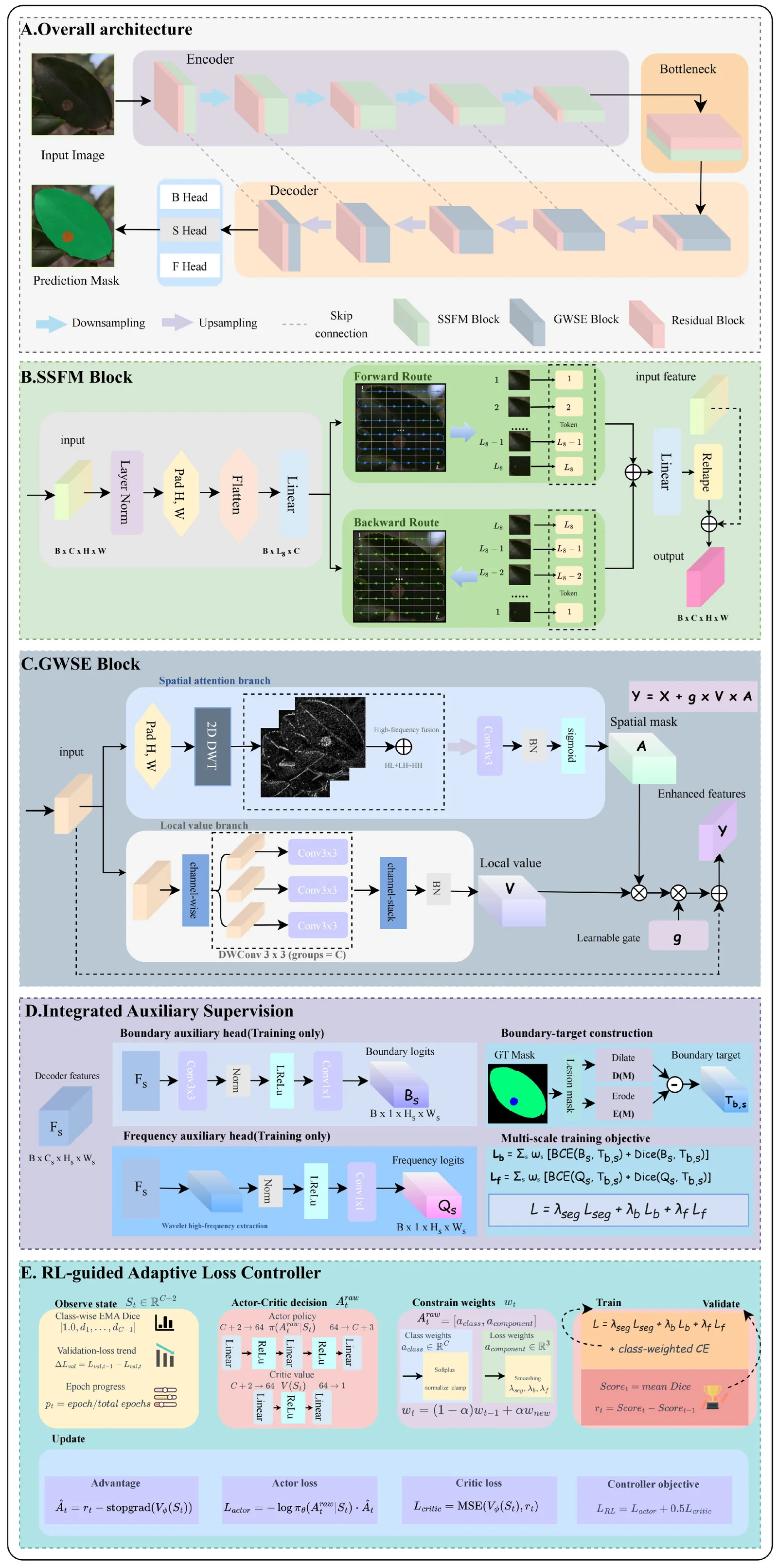
BFMambaNet Overall Framework.

As shown in Figure 3, the proposed framework contains five main parts.

1. **Overall architecture:** BFMambaNet adopts an encoder-decoder structure. The encoder extracts hierarchical features from the input image. The decoder progressively restores spatial resolution and produces the final segmentation mask through the segmentation head.
2. **SSFM block:** The Spatial Selective Feature Modeling block is designed as a Global Semantic Mamba block. It is derived from the selective state-space modeling principle of Mamba and the bidirectional visual sequence modeling idea of Mamba-2. It captures long-range lesion context through forward and backward semantic scanning.
3. **GWSE block:** The Gated Wavelet Spatial Enhancement block introduces wavelet-domain high-frequency information into feature refinement. It enhances weak lesion edges and fine pathological textures through a learnable spatial gate.
4. **Integrated auxiliary supervision:** Boundary and frequency auxiliary heads are attached to decoder features during training. The boundary target is generated from the ground-truth mask by dilation and erosion. Thus, the network receives explicit contour and high-frequency supervision without extra manual boundary labels.
5. **RL-guided adaptive loss controller:** The controller observes class-wise segmentation performance, validation-loss trend, and epoch progress. It dynamically adjusts class-wise reweighting factors and the weights of segmentation, boundary, and frequency losses. This helps reduce optimization bias caused by fixed loss settings and improves training stability.

#### 3.2.1. Overall Architecture

Given an input image $X\in R^{B\times C\times H\times W}$, the encoder first maps it into multi-scale features:

(1) $$\left\{F_{1},F_{2},\ldots ,F_{S}\right\}$$

To provide a comprehensive overview of the network hierarchy, the detailed configuration of BFMambaNet is presented in Table 2. Specifically, the network processes input patches of size 512×512 and consists of S=8 stages in actual implementation (note that Figure 3 illustrates a simplified 5-stage conceptual architecture for visual clarity). The spatial resolution progressively halves from 512×512 down to 4×4 at the bottleneck, while the channel capacity increases from a base of 32 up to a maximum of 512 channels. As shown in the diagram, the shallowest encoder stage consists solely of residual convolutional blocks, while all subsequent stages (Stages 2–8) are equipped with the Global Semantic Mamba-based SSFM block. This enables the encoder to learn both local disease appearance and global spatial dependency. Each residual block sequentially applies a 3×3 Convolution, Instance Normalization (IN), and LeakyReLU activation to preserve local texture information and stabilize gradient propagation. Downsampling between stages is achieved via strided convolution (stride of 2).

The decoder starts from the deepest feature map. At each stage, the low-resolution feature is upsampled using nearest neighbor interpolation followed by a 1×1 convolution. The upsampled feature is then fused with the corresponding skip feature from the encoder via channel-wise concatenation. Subsequently, a Gated Wavelet Spatial Enhancement (GWSE) block and a residual block are used to refine the fused representation. The final decoder output is sent to the segmentation head to predict the pixel-wise disease mask. During training, intermediate decoder features are also fed into the boundary head and frequency head. During inference, only the main segmentation output is used.

#### 3.2.2. Global Semantic Mamba-Based SSFM Block

Plant disease regions often have irregular shapes and scattered distributions. Some lesions are visually similar to leaf veins, shadows, or natural leaf textures. Therefore, local convolution alone may not provide enough semantic discrimination. To address this problem, we design the SSFM block as a Global Semantic Mamba (GSM) block.

For an input feature map(F), the block first applies LayerNorm along the channel dimension. The normalized feature is projected into a hidden semantic space. Then, two selective scanning routes are used. One route scans the spatial sequence in the forward direction. The other scans it in the backward direction. The two routes are fused as:

(2) $$F_{m}={\mathrm{Proj}}_{out}\left({\mathrm{GSM}}_{f}\left(F\right)+{\mathrm{GSM}}_{b}\left(F\right)\right)$$

Here, GSMf and GSMb denote the forward and backward Global Semantic Mamba routes. A residual connection is used to stabilize training:

(3) $$F_{out}=F+F_{m}$$

This design allows the network to capture long-range semantic dependency with efficient computation. It helps the model suppress isolated background responses and maintain coherent lesion structures.

#### 3.2.3. Gated Wavelet Spatial Enhancement Block

Lesion boundaries are often weak and low contrast. Small lesions may also lose details during repeated downsampling and upsampling. To preserve these details, we introduce the GWSE block.

Specifically, the 2D Haar DWT is performed in an intra-channel manner (i.e., independently across each channel). Given an input feature map $X\in R^{B\times C\times H\times W}$, the transform produces four sub-bands: one low-frequency component (LL) and three high-frequency components (HL, LH, HH):

(4) LL,HL,LH,HH=DWT(X)

Each sub-band has a tensor size of $R^{B\times C\times \frac{H}{2}\times \frac{W}{2}}$. To formulate the high-frequency spatial guidance, the three high-frequency sub-bands are directly summed:

(5) $$H_{freq}=HL+LH+HH$$

Although direct summation theoretically carries the risk of canceling out positive and negative edge responses in opposing directions, it is intentionally adopted to maintain computational efficiency, avoiding the 3C channel expansion that concatenation would require. Empirically, the direct signed summation acts as an unweighted superposition of multi-directional gradients. Because this aggregated signal is immediately fed into a subsequent learnable spatial attention branch, the convolutional filters can adaptively process these signed gradient distributions without suffering severe cancellation collapse.

To align the spatial dimensions, the aggregated high-frequency map $H_{freq}$ is then upsampled back to the original $R^{B\times C\times H\times W}$ resolution using bilinear interpolation. Subsequently, it passes through the spatial attention branch, which is constructed by a standard 3×3 convolution (padding = 1), Batch Normalization (BN), and a Sigmoid activation function, yielding a spatial attention mask *A* constrained between 0 and 1. In parallel, the local feature branch processes the original input *X* using a depthwise convolution (3×3, padding = 1, groups = *C*) followed by BN to preserve channel-wise local spatial details with minimal parameter overhead, yielding local features *V*. Finally, the attention mask modulates the local features, and the result is added back to the input via a learnable gating parameter α:

(6) Y=X+α·V·A

This gate α is initialized precisely to 0.0 without any explicit bounding constraints, ensuring that the entire GWSE block behaves as an identity mapping at the onset of training. This zero-initialization strategy prevents chaotic gradient fluctuations and stabilizes the early optimization dynamics of the network.

#### 3.2.4. Integrated Boundary-Frequency Auxiliary Supervision

To further improve structural fidelity, BFMambaNet introduces boundary-frequency auxiliary supervision during training. The boundary head predicts a single-channel boundary logit map from decoder features. Since manual boundary annotations are not available, the boundary target is automatically constructed from the ground-truth segmentation mask.

Given the lesion mask Y, we first extract the lesion region and then apply dilation and erosion:

(7) $$T_{b}=\mathrm{Dilate}\left(Y\right)-\mathrm{Erode}\left(Y\right)$$

The boundary auxiliary loss is defined as the combination of BCE and Dice loss:

(8) $$L_{b}=\mathrm{BCE}\left(B_{s},T_{b,s}\right)+\mathrm{Dice}\left(B_{s},T_{b,s}\right)$$

The frequency head predicts frequency logits from high-frequency decoder features. It is supervised by the same boundary target because lesion contours are strongly related to high-frequency responses:

(9) $$L_{f}=\mathrm{BCE}\left(Q_{s},T_{b,s}\right)+\mathrm{Dice}\left(Q_{s},T_{b,s}\right)$$

For multi-scale auxiliary outputs, the weight of each scale is defined as:

(10) $$\omega_{s}=\frac{1/2^{s}}{\sum_{s}1/2^{s}}$$

The auxiliary loss is:

(11) $$L_{aux}=\sum_{s}\omega_{s}\left(L_{b}^{s}+L_{f}^{s}\right)$$

The main segmentation loss combines cross-entropy and Dice loss:

(12) $$L_{seg}=L_{CE}+L_{Dice}$$

Therefore, the complete objective is:

(13) $$L=\lambda_{seg}L_{seg}+\lambda_{b}L_{b}+\lambda_{f}L_{f}$$

This design provides direct supervision for region consistency, contour accuracy, and high-frequency detail recovery.

#### 3.2.5. RL-Guided Adaptive Loss Controller

Fixed loss weights are not always suitable for plant disease segmentation. In the early training stage, the model usually needs stable region-level optimization to learn the basic distribution of leaves and lesions. In later stages, stronger boundary and frequency constraints are often required to refine weak contours and recover small pathological structures. Moreover, the pixel distribution of plant disease images is highly imbalanced. Background and healthy leaf regions occupy most pixels, whereas lesion regions are much smaller. This imbalance may cause the dominant classes to guide most gradients. To address this problem, BFMambaNet introduces an RL-guided adaptive loss controller, as shown in Part E of Figure 3.

At the end of epoch *t*, the controller observes the current training state. Following the formulation in the framework, the state is defined as:

(14) $$S_{t}\in R^{C+2}$$

(15) $$S_{t}=\left[d_{1},d_{2},\ldots ,d_{C},\Delta L_{val},p_{t}\right]$$

where $d_{c}$ denotes the class-wise exponential moving average (EMA) Dice score of class *c*. The validation-loss trend is calculated as:

(16) $$\Delta L_{val}=L_{val,t-1}-L_{val,t}$$

and the training progress is represented by:

(17) $$p_{t}=\frac{epoch}{total\;epochs}$$

Thus, the state vector simultaneously describes class-wise segmentation quality, validation optimization trend, and the current training stage. These observations provide the controller with compact but informative feedback for adaptive loss reweighting.

The actor network receives $S_{t}$ and outputs a raw action:

(18) $$A_{t}^{raw}=\pi_{\theta }\left(A_{t}^{raw}|S_{t}\right)$$

The raw action is divided into two parts:

(19) $$A_{t}^{raw}=\left[\alpha_{class},\alpha_{component}\right]$$

where $\alpha_{class}\in R^{C}$ denotes the class-wise raw action and $\alpha_{component}\in R^{3}$ denotes the component-wise raw action for the segmentation, boundary, and frequency objectives. The class-wise branch is transformed by Softplus, mean normalization, and clipping to obtain stable class reweighting factors:

(20) $$w_{c}=\mathrm{Clamp}\left(\frac{\mathrm{Softplus}(\alpha_{c})}{\mathrm{Mean}(\mathrm{Softplus}\left(\alpha_{class}\right))}\right)$$

These class factors are applied inside the cross-entropy term of $L_{seg}$ and are not treated as an additional loss item. The component-wise branch constrains the loss weights into predefined ranges. The constrained weights are denoted as $\lambda_{seg}$, $\lambda_{b}$, and $\lambda_{f}$. To prevent abrupt changes between adjacent updates, the generated weights are smoothed before being applied:

(21) $$w_{t}=\left(1-\alpha \right)w_{t-1}+\alpha w_{new}$$

During training, the adaptive objective follows the formulation in the framework:

(22) $$L_{train}=\lambda_{seg}L_{seg}+\lambda_{b}L_{b}+\lambda_{f}L_{f}$$

where the class-wise factors are embedded in the cross-entropy part of $L_{seg}$, and the three component weights $\lambda_{seg}$, $\lambda_{b}$, and $\lambda_{f}$ are guided by $\alpha_{component}$. Through this design, the controller adjusts both class-wise learning emphasis and the relative importance of segmentation, boundary, and frequency supervision during training.

After validation, the controller computes the reward according to the change in validation score. Consistent with the framework, the score is defined by mean Dice:

(23) $$Score_{t}=mean\;Dice$$

The reward is then calculated as the immediate performance delta:

(24) $$r_{t}=Score_{t}-Score_{t-1}$$

A positive reward indicates that the current weighting strategy improves validation segmentation performance, while a negative reward suggests that the previous action should be penalized. It is crucial to note that our formulation deliberately models the dynamic loss weighting problem as a **Contextual Bandit** (or equivalently, an Actor-Critic method with a discount factor γ=0), rather than a standard Markov Decision Process (MDP) with long-term cumulative returns. This design is theoretically motivated by the severe credit assignment problem in deep learning optimization: the “environment” (the network’s loss landscape) is highly non-stationary, and validation metrics are heavily influenced by stochastic mini-batch sampling and optimizer momentum. Attempting to optimize a long-term temporal difference (TD) target across dozens of epochs introduces massive variance and credit-assignment noise. Therefore, the controller focuses solely on maximizing the immediate expected improvement given the current training state context.

Accordingly, the critic network estimates the expected immediate reward of the current state, rather than a cumulative return:

(25) $$V_{\phi }\left(S_{t}\right)$$

The advantage function is computed purely based on the immediate reward without a future value term:

(26) $${\hat{A}}_{t}=r_{t}-\mathrm{stopgrad}\left(V_{\phi }\left(S_{t}\right)\right)$$

The actor loss is defined as:

(27) $$L_{actor}=-\log\pi_{\theta }\left(A_{t}^{raw}|S_{t}\right)\cdot {\hat{A}}_{t}$$

and the critic loss is strictly fitted to the immediate reward $r_{t}$ rather than a TD target:

(28) $$L_{critic}=\mathrm{MSE}\left(V_{\phi }\left(S_{t}\right),r_{t}\right)$$

Finally, the optimization objective of the controller is:

(29) $$L_{RL}=L_{actor}+0.5L_{critic}$$

Through this actor–critic optimization process, the controller learns how to adjust class-wise reweighting and segmentation, boundary, and frequency supervision according to the training state. This adaptive mechanism improves optimization stability and helps BFMambaNet generate sharper and more complete lesion masks under complex field conditions. 

## 4. Results and Discussion

To ensure the reproducibility of our results, all evaluations in this study were conducted under a uniform hardware and software environment. This standardized platform not only improves training efficiency but also provides a fair baseline for comparing different algorithms. The specific computing equipment and experimental hyperparameters are summarized in Table 3 and Table 4, respectively.

To ensure training stability and prevent the meta-controller from being misled by chaotic gradients during the early stages of optimization, a specialized training preheating scheme is implemented for the RL-guided adaptive loss controller. During the static warm-up period (Epochs 0 to 19), the RL controller remains silent without predicting any action weights or updating its policy gradients. The main segmentation network is trained using default configurations: uniform weights of 1.0 for all disease classes, and static component weights of 1.0 for the primary segmentation loss, 0.1 for the boundary auxiliary loss, and 0.0 for the remaining auxiliary losses. This allows the network backbone to establish baseline feature representations first. From Epoch 20 onwards, the dynamic adjustment phase is activated. Every 5 epochs, the controller receives the class-wise validation Dice scores as state inputs and invokes its policy to output new class-wise and component-wise loss weights. These weights are updated using a momentum smoothing factor of 0.3 with the previous weights to prevent abrupt weight updates. Concurrently, the difference between the current validation score and the previous score is calculated as the reward to update the parameters of the Actor-Critic meta-controller via policy gradients.

### 4.1. Evaluation Index

To comprehensively evaluate the segmentation performance of the proposed model, we used five standard metrics derived from the confusion matrix: Precision, Recall, Intersection over Union (IoU), mean Intersection over Union (mIoU), and the Dice coefficient. In this context, true positives (TP) indicate pixels that are correctly identified as target regions, and true negatives (TN) denote background pixels that are correctly classified as non-target regions. False positives (FP) correspond to background pixels incorrectly predicted as target regions, whereas false negatives (FN) represent target pixels that the model fails to detect.

Precision is defined as the ratio of correctly predicted target pixels to all pixels predicted as the target. It reflects the reliability of positive predictions and is formulated as:

(30) $$\mathrm{Precision}=\frac{TP}{TP+FP}$$

Recall measures the proportion of actual target pixels that are correctly identified. It reflects the ability of the model to capture complete lesion regions and is formulated as:

(31) $$\mathrm{Recall}=\frac{TP}{TP+FN}$$

The Intersection over Union (IoU) measures the spatial overlap between the predicted segmentation mask and the corresponding ground-truth mask for a given class. It is calculated as:

(32) $$\mathrm{IoU}=\frac{TP}{TP+FP+FN}$$

The mean Intersection over Union (mIoU) is used to evaluate the global segmentation accuracy by averaging the IoU values over all semantic categories. Given *N* classes, mIoU is defined as:

(33) $$\mathrm{mIoU}=\frac{1}{N}\sum_{i=1}^{N}{\mathrm{IoU}}_{i}$$

Finally, the Dice coefficient is introduced to measure the structural similarity between the predicted and actual target regions. Considering the imbalance between lesion pixels and background pixels in the dataset, Dice provides an effective metric for evaluating the model’s ability to fit fine-grained lesion boundaries. It is calculated as:

(34) $$\mathrm{Dice}=\frac{2TP}{2TP+FP+FN}$$

Although conventional metrics such as Precision, Recall, Dice, and mIoU are widely used, they are primarily sensitive to global region overlap and fail to provide specialized quantitative support for boundary precision. Therefore, we introduce Boundary Intersection over Union (Boundary IoU) and Normalized Surface Distance (NSD) as auxiliary evaluation metrics to quantitatively assess the accuracy of model predictions along the lesion contours. Boundary IoU focuses exclusively on the quality of boundary segmentation by calculating the overlap of predictions and ground truths within a contour distance threshold *d*, and is formulated as:

(35) $$\begin{array}{c} \mathrm{Boundary}\;\mathrm{IoU}=\frac{|\left(G_{d}\cap G\right)\cap \left(P_{d}\cap P\right)|}{|\left(G_{d}\cap G\right)\cup \left(P_{d}\cap P\right)|} \end{array}$$

where *G* and *P* denote the ground truth and predicted segmentation mask, respectively, and $G_{d}$ and $P_{d}$ represent the boundary pixels within a distance threshold *d* (set to 1 or 2 pixels in this study) from the respective contours. Similarly, NSD measures the agreement between boundary surfaces within a tolerance threshold τ. It evaluates the proportion of boundary points on one surface that lie within a distance of τ to the other surface, and is defined as:

(36) $$\begin{array}{c} \mathrm{NSD}=\frac{|B_{G}\cap B_{P,\tau }\left|+\right|B_{P}\cap B_{G,\tau }|}{|B_{G}\left|+\right|B_{P}|} \end{array}$$

where $B_{G}$ and $B_{P}$ represent the boundary pixel sets of ground truth and prediction, while $B_{P,\tau }$ (or $B_{G,\tau }$) denotes the set of boundary pixels whose distance to the other boundary is within the tolerance threshold τ.

### 4.2. Comparison with State-of-the-Art Methods

To evaluate the effectiveness of the proposed BFMambaNet, we compared it with several representative semantic segmentation frameworks, including CNN-based models, Transformer-based models, and Mamba-based models. The CNN-based baselines include DeepLabV3, which uses atrous convolution to enlarge the receptive field [20]; DeepLabV3+, which further introduces an encoder–decoder structure with atrous separable convolution [56]; and UNet, which is a classical encoder–decoder segmentation network with skip connections [57]. The Transformer-based baselines include SegFormer, which combines a hierarchical Transformer encoder with a lightweight decoder [41], and OneFormer, which formulates different segmentation tasks with a unified Transformer architecture [58]. PSPNet, which aggregates pyramid-level contextual information for scene parsing [59], and HRNet, which maintains high-resolution representations throughout feature extraction [60], are also included as representative context-aggregation and high-resolution segmentation baselines. Mask2Former is further included because it uses masked attention for universal image segmentation [61]. In addition, VMamba is selected as the Mamba-based baseline because it introduces selective state-space modeling into visual feature learning [25]. Furthermore, SegMamba [62] and H-vmunet [63] are included as representative visual Mamba architectures designed to model visual sequences and capture hierarchical biomedical segmentation structures, respectively. MedSAM [64] is also incorporated as a representative foundation model adapted for general medical and fine-grained image segmentation. This baseline setting allows a fair comparison with local convolutional models, attention-based global models, state-space-based global models, and foundation-based segmentation frameworks. The quantitative results are shown in Table 5. BFMambaNet achieves the best overall performance among all compared methods, with a Precision of 92.39%, Recall of 91.43%, Dice of 91.26%, and mIoU of 85.46%. Compared with VMamba, BFMambaNet improves the mIoU from 83.42% to 85.46%, indicating that the proposed boundary-frequency guidance and adaptive optimization strategy further enhance the segmentation capability of the Mamba-based framework.

**Table 5:** Performance evaluation of different *Camellia oleifera* disease segmentation frameworks. The highest value is highlighted in bold.

Methods	Precision (%)	Recall (%)	Dice (%)	mIoU (%)	Boundary IoU (%)	NSD (%)
HRNet	84.69 ± 0.27	83.70 ± 0.41	83.17 ± 0.32	73.72 ± 0.35	72.07 ± 0.44	74.34 ± 0.32
PSPNet	89.15 ± 0.40	88.36 ± 0.38	88.00 ± 0.45	80.99 ± 0.23	78.88 ± 0.21	81.32 ± 0.21
SegFormer	89.61 ± 0.21	89.17 ± 0.39	89.64 ± 0.36	81.54 ± 0.37	79.74 ± 0.46	81.86 ± 0.34
DeepLabV3	87.81 ± 0.47	88.16 ± 0.20	88.89 ± 0.21	80.02 ± 0.27	77.86 ± 0.21	80.24 ± 0.24
DeepLabV3+	89.58 ± 0.31	89.59 ± 0.50	89.65 ± 0.24	81.23 ± 0.31	79.50 ± 0.44	81.66 ± 0.40
UNet	89.22 ± 0.37	86.61 ± 0.44	87.17 ± 0.29	78.35 ± 0.50	76.61 ± 0.47	78.99 ± 0.25
OneFormer	87.67 ± 0.40	90.53 ± 0.34	88.77 ± 0.37	80.39 ± 0.49	78.55 ± 0.33	80.68 ± 0.40
Mask2Former	89.31 ± 0.52	89.28 ± 0.20	89.39 ± 0.45	81.62 ± 0.31	79.79 ± 0.25	81.96 ± 0.20
VMamba	91.01 ± 0.51	89.81 ± 0.43	90.04 ± 0.27	83.42 ± 0.33	81.68 ± 0.42	84.20 ± 0.40
SegMamba	90.40 ± 0.48	89.54 ± 0.34	89.61 ± 0.29	82.04 ± 0.26	80.30 ± 0.25	82.54 ± 0.24
H-vmunet	88.73 ± 0.48	87.90 ± 0.45	88.14 ± 0.25	80.62 ± 0.49	78.91 ± 0.38	80.90 ± 0.47
MedSAM	89.04 ± 0.25	90.25 ± 0.42	89.06 ± 0.20	81.62 ± 0.42	79.85 ± 0.23	82.37 ± 0.33
BFMambaNet	**92.39 ± 0.48**	**91.43 ± 0.24**	**91.26 ± 0.28**	**85.46 ± 0.23**	** 83.91 ± 0.35 **	** 86.36 ± 0.34 **

The class-wise IoU comparison is presented in Table 6. BFMambaNet obtains the best IoU on Anthracnose, Worm Holes, Red Spot, Soft Rot, and Algae Spot. These categories usually contain irregular lesion shapes, weak boundary transitions, or visual patterns similar to healthy leaf textures. The improvement demonstrates that the proposed framework is effective for distinguishing subtle pathological regions from complex field backgrounds. Although several baseline models perform well on individual categories, BFMambaNet shows a more balanced performance across different disease types.

**Table 6:** Class-wise IoU comparison of different segmentation models. The highest IoU value in each category is highlighted in bold.

Methods (IoU%)	Leaf	White Scab	Anthra Cnose	Worm Holes	Red Spot	Sooty Mold	Soft Rot	Algae Spot
HrNet	80.59 ± 0.44	70.75 ± 0.33	66.13 ± 0.21	59.81 ± 0.50	82.30 ± 0.29	82.98 ± 0.24	89.36 ± 0.39	67.68 ± 0.51
PSPNet	91.37 ± 0.46	72.47 ± 0.22	70.90 ± 0.44	69.91 ± 0.47	83.81 ± 0.33	88.88 ± 0.22	87.66 ± 0.39	71.07 ± 0.35
Segformer	93.49 ± 0.36	65.03 ± 0.30	73.50 ± 0.46	69.37 ± 0.27	88.29 ± 0.42	83.99 ± 0.35	86.03 ± 0.41	82.96 ± 0.44
DeeplabV3	92.06 ± 0.20	66.12 ± 0.29	70.65 ± 0.43	66.30 ± 0.18	89.30 ± 0.36	82.76 ± 0.31	88.29 ± 0.42	78.11 ± 0.34
DeeplabV3+	**93.75 ± 0.23**	66.02 ± 0.20	71.21 ± 0.21	68.06 ± 0.22	90.03 ± 0.32	82.79 ± 0.30	88.17 ± 0.38	76.88 ± 0.25
UNet	89.76 ± 0.38	68.79 ± 0.27	69.37 ± 0.52	66.85 ± 0.27	83.88 ± 0.52	76.66 ± 0.18	85.55 ± 0.40	76.55 ± 0.46
OneFormer	90.28 ± 0.33	71.14 ± 0.51	71.05 ± 0.30	69.26 ± 0.48	84.44 ± 0.42	90.13 ± 0.31	86.40 ± 0.23	78.18 ± 0.27
Mask2Former	91.34 ± 0.23	**74.94 ± 0.22**	73.77 ± 0.38	69.35 ± 0.34	85.19 ± 0.21	**91.29 ± 0.19**	87.19 ± 0.21	69.45 ± 0.37
Vmamba	91.11 ± 0.44	71.50 ± 0.50	75.14 ± 0.48	72.68 ± 0.47	86.65 ± 0.45	90.45 ± 0.43	86.81 ± 0.19	75.82 ± 0.37
SegMamba	91.34 ± 0.38	70.17 ± 0.36	74.82 ± 0.38	71.21 ± 0.43	87.65 ± 0.28	88.00 ± 0.37	87.20 ± 0.38	79.29 ± 0.33
H-vmunet	90.62 ± 0.24	69.55 ± 0.35	71.38 ± 0.40	68.31 ± 0.37	86.72 ± 0.32	87.54 ± 0.34	86.87 ± 0.39	76.12 ± 0.31
MedSAM	91.26 ± 0.46	71.11 ± 0.49	73.34 ± 0.37	71.06 ± 0.31	86.96 ± 0.25	88.49 ± 0.29	86.72 ± 0.27	74.53 ± 0.44
BFMambaNet	92.15 ± 0.20	72.83 ± 0.25	**80.61 ± 0.47**	**74.42 ± 0.36**	**90.57 ± 0.41**	90.35 ± 0.26	**90.22 ± 0.26**	**85.78 ± 0.28**

**Figure 4:**
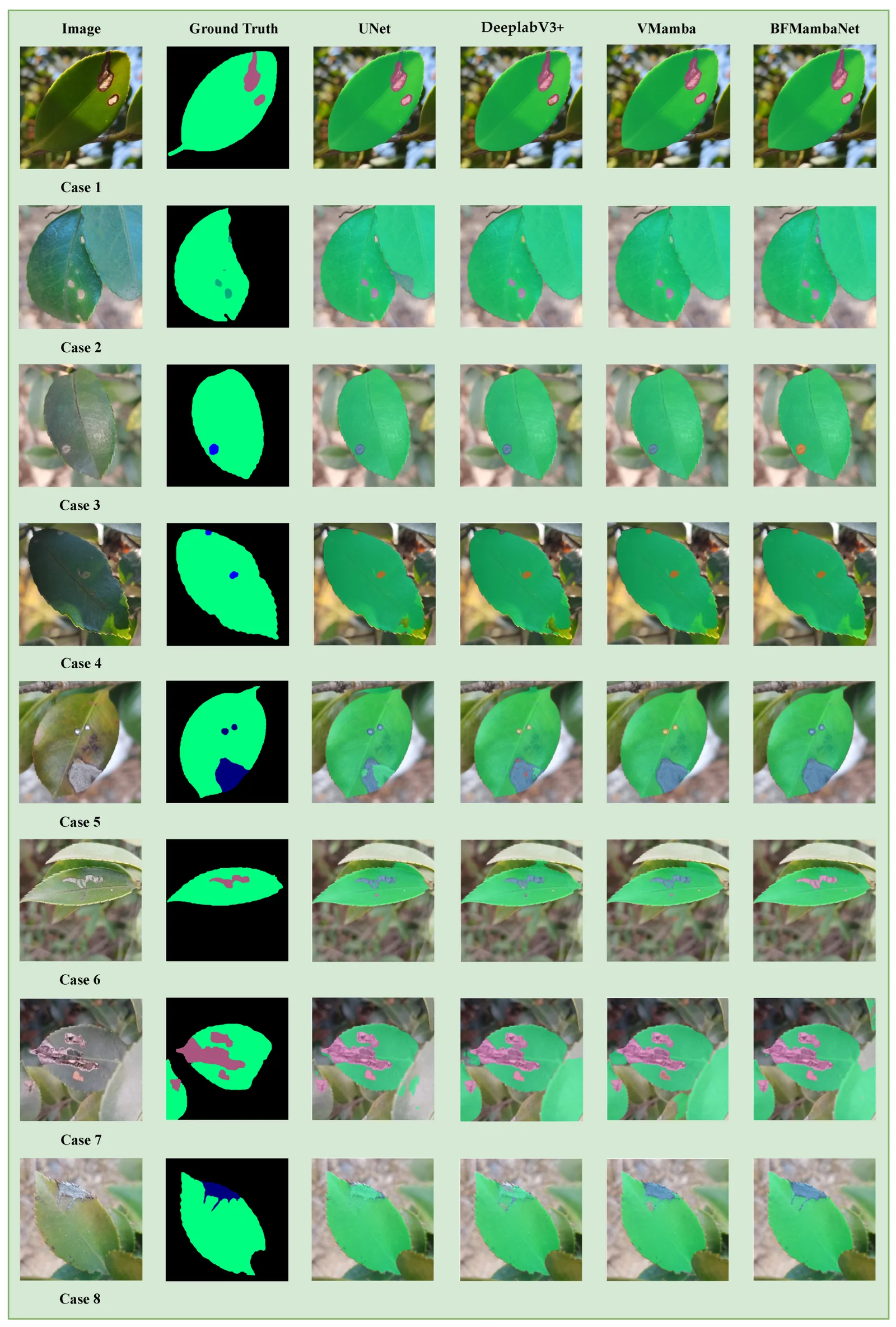
Qualitative comparison of segmentation results under complex field conditions. The figure compares the original images, ground-truth masks, and predictions generated by representative baseline models and BFMambaNet.

To further examine the visual quality of different models, we present qualitative segmentation results in Figure 4. The figure compares the original image, ground truth, and predictions of UNet, DeepLabV3+, VMamba, and BFMambaNet. The selected cases cover representative field conditions with uneven illumination, complex leaf texture, weak lesion boundaries, and background interference. These cases are difficult because disease regions may show low contrast, irregular shapes, and visual patterns similar to healthy leaf tissues.

As shown in Figure 4, the baseline models can generally locate the main leaf region, but their lesion predictions are still affected by illumination variation, leaf texture, and weak boundaries. In Case 1, part of the leaf surface is under shadow, which weakens the contrast between the lesion and the surrounding tissue. UNet and DeepLabV3+ show less stable lesion coverage, and VMamba still produces slight boundary deviation. In contrast, BFMambaNet accurately preserves the lesion region under the shaded condition and gives a prediction closer to the ground truth. In Case 2, the lesion appears near the leaf edge and forms a hole-like structure. This region is easily confused with the leaf boundary or background. BFMambaNet still segments this edge hole more completely, while the compared models tend to miss part of the target or generate less accurate contours.

Case 3 further shows the advantage of BFMambaNet in category discrimination. Algae Leaf Spot has visual characteristics that are close to Anthracnose, especially when the spot is dark and the boundary is not clear. The baseline models fail to provide a precise category-level prediction in this case. BFMambaNet produces the most accurate segmentation for Algae Leaf Spot, indicating that the proposed global semantic modeling helps distinguish visually similar disease patterns. In Case 4, the specular highlight region at the lower part of the leaf introduces strong visual interference. This makes the lesion area difficult to separate from the leaf surface. The compared models are affected by the bright region and produce incomplete or biased predictions, whereas BFMambaNet still identifies the target region more accurately. In Case 5, the disease region is located close to the lower leaf area, where shadows and natural color changes make the boundary less clear. UNet and DeepLabV3+ show obvious category confusion around the lower lesion, and VMamba produces a smoother but less complete response. BFMambaNet better preserves the main diseased area and keeps the small isolated spot on the upper part of the leaf. This indicates that the proposed model can handle local texture changes without losing small lesion cues.

In Case 6, the lesion appears as a narrow and elongated structure along the leaf surface. Such a shape is easy to break into discontinuous fragments or to be absorbed into the leaf background. The baseline models detect the approximate location, but the predicted color and shape are not stable. BFMambaNet generates a more continuous lesion mask and follows the thin lesion distribution more closely. In Case 7, the disease area has an irregular boundary and a large intensity variation. The lower part of the image also contains nearby leaf regions, which can introduce false foreground responses. The compared models tend to over-expand the lesion or generate noisy predictions near the surrounding leaf area. BFMambaNet gives a cleaner mask and better preserves the irregular contour of the target region. In Case 8, the lesion is close to the leaf edge, and the upper boundary is affected by the serrated leaf margin and background texture. This makes the disease boundary difficult to separate from the natural leaf edge. BFMambaNet produces a more complete prediction along the upper lesion boundary, while the baseline models either miss part of the edge region or produce rough contours. These visual results are consistent with the quantitative improvements in Table 5 and Table 6. They indicate that global semantic modeling, wavelet-guided spatial enhancement, and boundary-frequency supervision work together to improve segmentation robustness under complex field conditions.

### 4.3. Ablation Study

The ablation study is used to analyze the contribution of each key component in BFMambaNet. By comparing different module combinations, the effects of global semantic Mamba modeling, gated wavelet spatial enhancement, boundary-frequency auxiliary supervision, and RL-guided adaptive loss control can be evaluated separately. This experiment helps verify whether the performance improvement is caused by a single module or by the complementary interaction among multiple components.

Table 7 reports the quantitative contribution of each main component. The baseline U-Mamba obtains a Dice of 88.03% and an mIoU of 81.73%. After adding SSFM, Dice increases by 0.64% and mIoU increases by 0.98%. This indicates that global semantic modeling helps the network connect scattered lesion regions and suppress isolated background responses. GWSE also brings a clear gain. It improves Dice to 88.64% and mIoU to 83.19%. This result shows that wavelet-guided enhancement can recover weak lesion boundaries and fine pathological textures. When SSFM and GWSE are used together, Dice reaches 89.76% and mIoU reaches 84.43%. The joint improvement is larger than using either module alone. This suggests that long-range semantic context and local high-frequency details provide complementary information.

**Table 7:** Ablation study of the main components in BFMambaNet.

Method	SSFM	GWSE	B Head	F Head	RL Loss	Dice ↑	mIoU ↑
Baseline U-Mamba						88.03 ± 0.38	81.73 ± 0.31
+SSFM	✓					88.67 ± 0.37	82.71 ± 0.29
+GWSE		✓				88.64 ± 0.49	83.19 ± 0.28
+SSFM + GWSE	✓	✓				89.76 ± 0.48	84.43 ± 0.31
+Boundary head	✓	✓	✓			90.50 ± 0.21	84.54 ± 0.48
+Frequency head	✓	✓		✓		90.94 ± 0.48	84.55 ± 0.50
+B/F heads	✓	✓	✓	✓		90.54 ± 0.38	85.15 ± 0.36
Full model	✓	✓	✓	✓	✓	91.26 ± 0.28	85.46 ± 0.23

The auxiliary branches further refine the model after SSFM and GWSE. Adding the boundary head increases Dice from 89.76% to 90.50%. This shows that explicit contour supervision helps reduce boundary displacement. Adding the frequency head improves mIoU from 84.43% to 84.55%, which indicates better recovery of high-frequency lesion details. When the boundary and frequency heads are used together, Dice and mIoU reach 90.54% and 85.15%, respectively. The full model achieves the best result, with Dice of 91.26% and mIoU of 85.46%. Compared with the baseline, the full model improves Dice by 3.23% and mIoU by 3.73%. These results show that the final improvement is not produced by a single module. It comes from the joint effect of semantic modeling, wavelet enhancement, auxiliary supervision, and adaptive loss control.

**Figure 5:**
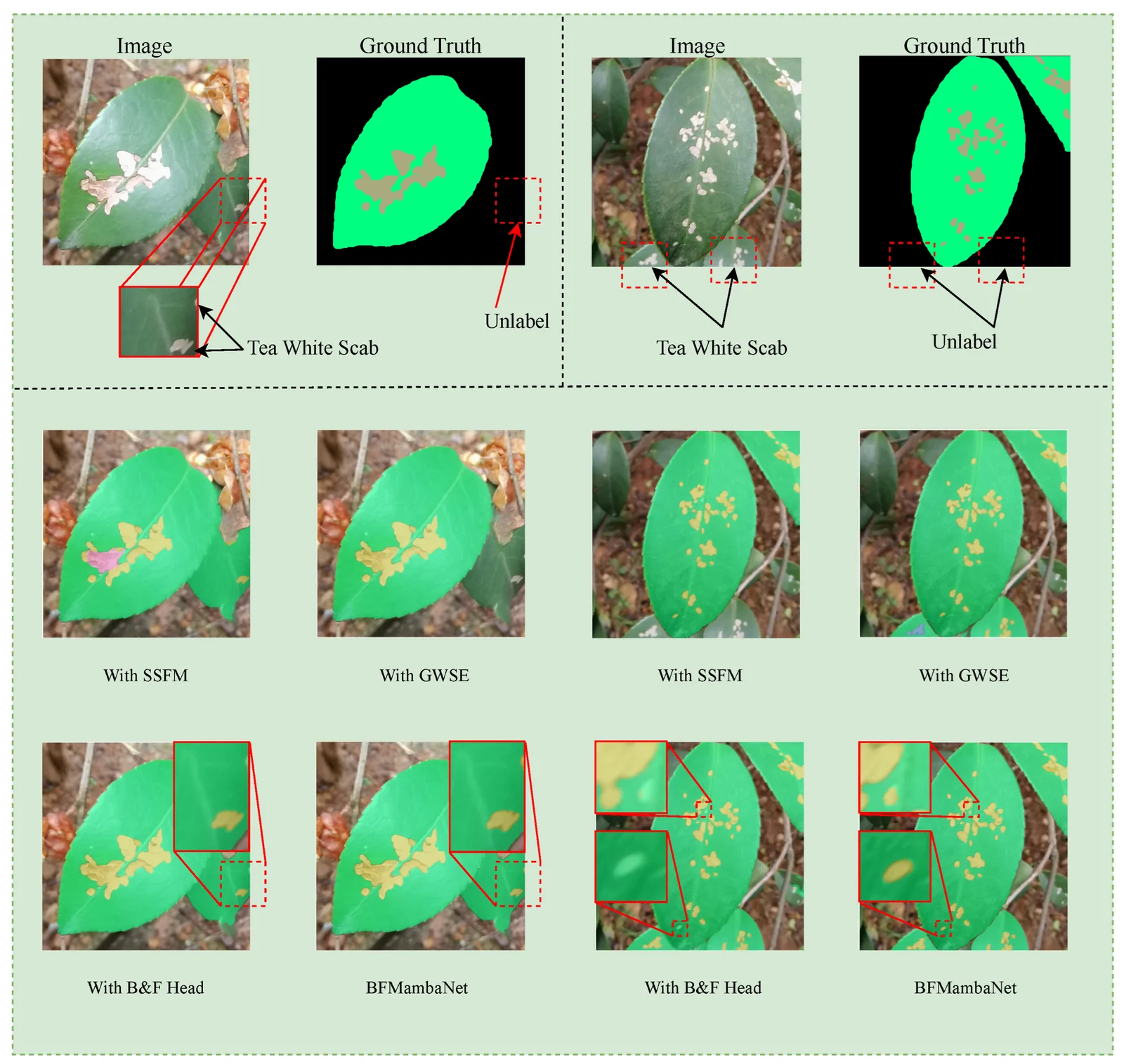
Qualitative visualization of ablation variants on disease-like regions that are visible in the image but absent from the manual annotation. The compared variants are arranged as With SSFM, With GWSE, With B&F Head, and BFMambaNet. Red dashed boxes indicate unlabelled pathological regions.

Figure 5 provides a qualitative analysis of a special annotation-related situation. In the two examples, some visible Tea White Scab regions are not included in the ground-truth mask, as marked by the red dashed boxes. These regions are visually similar to the labelled disease areas, but they are treated as background in the annotation. Therefore, predictions on these regions may be counted as false positives by strict pixel-level metrics. However, from the image content, they reflect whether the model can still respond to real but unlabelled pathological cues.

In the left example, a small white scab region appears near the lower-right leaf edge, but it is not annotated in the mask. With SSFM, the model outlines the central diseased region, but the prediction still contains incorrect segmentation around the labelled lesion and fails to respond clearly to the unlabelled edge spot. With GWSE, the boundary of the main annotated region becomes smoother and the texture response on the leaf surface is more stable; however, the lower-right unlabelled disease spot is still not captured. After introducing the B&F Head, the model produces a visible response to the unlabelled disease region, but it still fails to capture the most difficult tiny lesion located close to the leaf edge. BFMambaNet further recovers this edge lesion cue while producing a more compact edge-region prediction and a cleaner contour around the main lesion. In the right example, several small disease-like spots appear near the lower leaf region and around the main lesion cluster. The full model preserves these small responses more compactly than the partial variants. This suggests that the combination of global semantic modeling, wavelet-guided enhancement, and adaptive supervision improves the model’s ability to recognize subtle pathological patterns, even when some disease pixels are missing from the manual annotation.

Table 8 further analyzes the internal design of the GWSE block. When GWSE is removed, the model obtains a Dice of 88.75% and an mIoU of 83.67%. This result is lower than all GWSE variants, showing that high-frequency enhancement is useful for plant disease segmentation. The spatial branch alone improves Dice to 90.03% and mIoU to 84.30%. This indicates that the wavelet-guided spatial response helps the model locate weak lesion edges and suppress part of the background interference. The local value branch alone gives a smaller improvement, with Dice of 88.94% and mIoU of 84.00%. This suggests that local convolutional value features can refine regional texture, but they are not sufficient to recover ambiguous boundaries without explicit frequency guidance.

When the spatial branch and local value branch are combined without the learnable gate, Dice increases to 90.77% and mIoU increases to 84.95%. This shows that the two branches are complementary. The spatial branch provides boundary-sensitive attention, while the local value branch preserves local disease appearance. However, direct fusion may still introduce redundant or noisy responses from leaf veins, highlights, and background textures. After adding the learnable gate, the full GWSE block achieves the best Dice of 91.26% and an mIoU of 85.46%. The gate controls the strength of high-frequency enhancement and allows the network to select useful details more adaptively. Therefore, the full GWSE design is more stable than using either branch alone or using ungated fusion.

**Table 8:** Internal ablation study of the GWSE block.

Variant	Spatial Branch	Local Value Branch	Frequency Fusion	Learnable Gate	Dice ↑	mIoU ↑
w/o GWSE					88.75 ± 0.27	83.67 ± 0.20
Spatial only	✓		✓		90.03 ± 0.27	84.30 ± 0.48
Local only		✓			88.94 ± 0.32	84.00 ± 0.19
Spatial + Local, no gate	✓	✓	✓		90.77 ± 0.31	84.95 ± 0.26
Full GWSE	✓	✓	✓	✓	91.26 ± 0.28	85.46 ± 0.23

Table 9 evaluates the RL-guided adaptive loss controller. The fixed-weight setting corresponds to the model with SSFM, GWSE, and both auxiliary heads, but without RL adaptation. It obtains Dice of 90.52% and mIoU of 84.80%. With RL-CW only, Dice increases to 90.67% and mIoU increases to 85.27%. This shows that class-wise raw actions help the model reduce the bias caused by uneven category learning. With RL-LW only, Dice and mIoU further improve to 90.79% and 85.20%. This indicates that dynamically adjusting the weights of segmentation, boundary, and frequency losses is more directly related to multi-objective training stability. The full RL setting obtains the best performance, with Dice of 91.26% and mIoU of 85.46%. This result shows that class-level regulation and loss-component regulation are complementary. Together, they help the model balance region prediction, boundary refinement, and frequency-detail recovery during training.

**Table 9:** Ablation study of the RL-guided adaptive loss controller.

Variant	Fixed Weights	RL-CW	RL-LW	Dice ↑	mIoU ↑
Fixed weights	✓			90.52 ± 0.21	84.80 ± 0.30
RL-CW only		✓		90.67 ± 0.31	85.27 ± 0.34
RL-LW only			✓	90.79 ± 0.43	85.20 ± 0.45
Full RL		✓	✓	91.26 ± 0.28	85.46 ± 0.23

**Figure 6:**
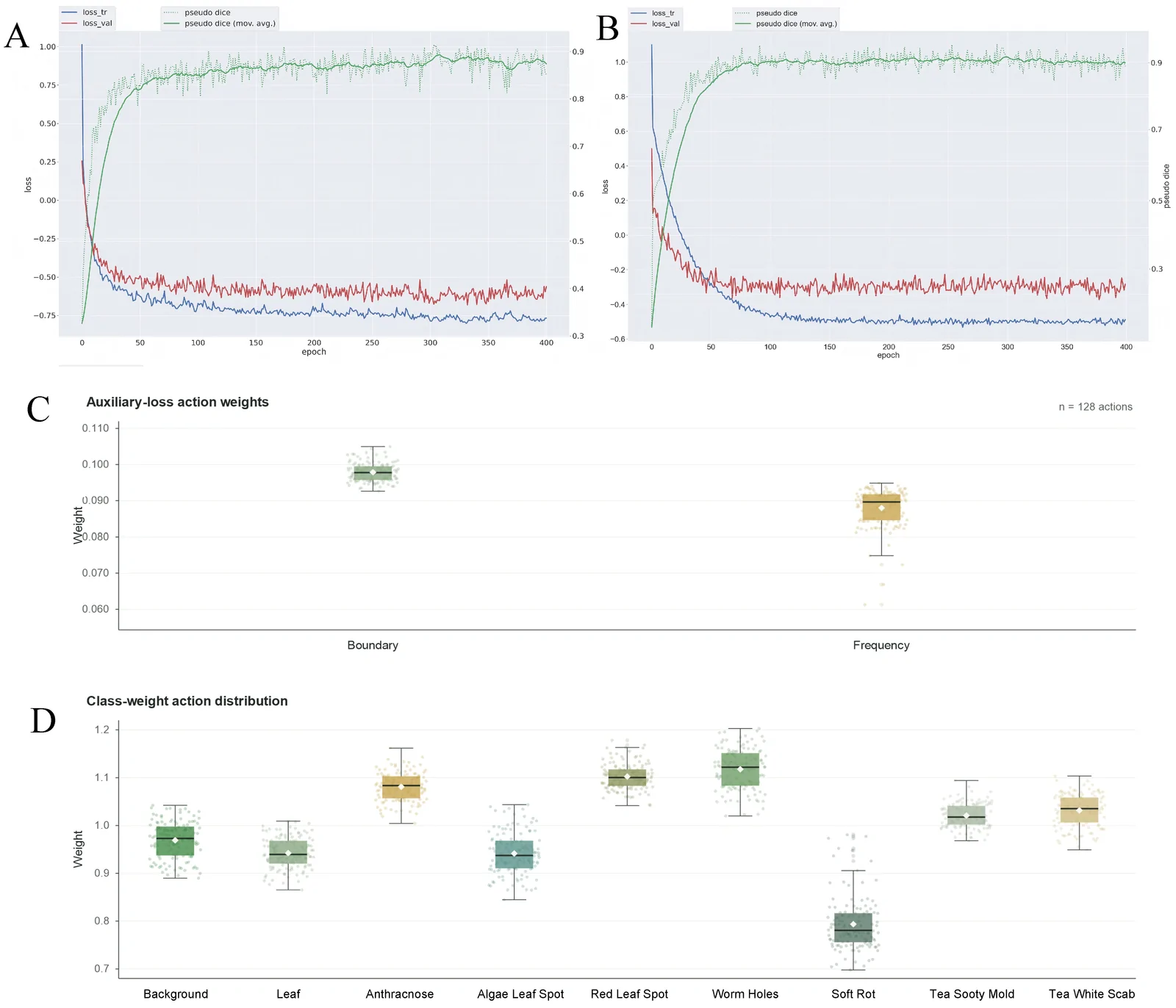
Analysis of training dynamics and adaptive loss reweighting. (**A**) Training and validation loss curves of the baseline U-Mamba. (**B**) Training and validation loss curves of the proposed BFMambaNet. (**C**) Statistical distribution of auxiliary loss weights ($\lambda_{\mathrm{boundary}}$ and $\lambda_{\mathrm{frequency}}$) outputted by the RL-guided adaptive loss controller during training. (**D**) Statistical distribution of class-wise loss weights ($w_{c}$) dynamically regulated by the meta-controller.

To further evaluate the optimization stability and training dynamics, we visualized the training and validation loss curves, alongside the adjustments of the reinforcement learning (RL) loss component weights and class-wise weights, as illustrated in Figure 6. As shown in Figure 6A, the baseline U-Mamba exhibits slower convergence and stabilizes at a higher training and validation loss. In contrast, BFMambaNet (Figure 6B) exhibits a faster convergence rate and reaches a lower, more stable loss. This enhanced convergence behavior is primarily attributed to the RL-guided meta-controller, which replaces static, suboptimal loss weighting with dynamic optimization paths. The validation loss curve shown in Figure 6B of BFMambaNet is also smoother than that of U-Mamba in Figure 6A, indicating that the smoothing mechanism applied to the Actor-Critic output prevents abrupt weight updates and stabilizes the gradient propagation throughout the training phase.

Figure 6C illustrates the statistical distribution of the two auxiliary loss weights ($\lambda_{\mathrm{boundary}}$ and $\lambda_{\mathrm{frequency}}$) dynamically outputted by the controller during training. The boxplot reveals that the RL controller allocates a higher average weight to the boundary auxiliary loss ($\lambda_{\mathrm{boundary}}$ median ≈0.098) than to the frequency auxiliary loss ($\lambda_{\mathrm{frequency}}$ median ≈0.088). This weighting strategy suggests that the model prioritizes boundary alignment during training to resolve the boundary ambiguity problem. By adaptively balancing these two auxiliary components, the RL controller effectively aligns the training objective with the model’s actual optimization needs, explaining the overall performance gains reported in Table 9.

Beyond loss components, the meta-controller also dynamically regulates the class-wise loss weights ($w_{c}$) to combat severe class imbalance and varying learning difficulties, as illustrated in the statistical boxplot of Figure 6D. Statistical analysis of the outputted class weights reveals a highly rational adaptive weighting mechanism. First, the controller assigns a high loss weight to underrepresented categories. For instance, the minority class Anthracnose, which accounts for only 10.2% of the dataset, receives an average weight of 1.0810 (median 1.0835), placing it in the highest weight tier. Similarly, Red Leaf Spot (15.2% proportion) and Worm Holes (18.1% proportion) receive high average weights of 1.1176 and 1.1024, respectively. This heavy penalization prevents rare pathological features from being overwhelmed by background gradients. Second, the controller restricts majority classes from dominating the optimization process. The majority class Tea White Scab, representing 42.1% of the dataset, receives a moderate average weight of 1.0315, which is lower than the weights assigned to the rare classes. Third, the controller dynamically compresses the weights of easy-to-segment categories. For example, the weight for Soft Rot (15.4% proportion) is compressed to an average of 0.7937 (median 0.7807) because its large lesions and distinct borders are easily learned by the network early on. By reducing the optimization focus on these easy classes, the RL agent shifts the model’s capacity to resolve more challenging, underrepresented symptoms.

This class-wise adaptive reweighting is strongly corroborated by the class-wise IoU improvements presented in Table 6. As shown in the table, the minority class Anthracnose exhibits a significant IoU increase from 75.14% (VMamba) to 80.61% (+5.47%). Similarly, the hard-to-segment category Algae Leaf Spot (15.4% proportion) experiences a massive IoU boost from 75.82% to 85.78% (+9.96%). For the majority class Tea White Scab, the IoU rises from 71.50% to 72.83% (+1.33%). Even for Soft Rot, whose weight was compressed to 0.7937, the IoU still improves from 86.81% to 90.22% (+3.41%), confirming that the network maintains a high-quality segmentation boundary while successfully reallocating gradient resources. These results validate that the RL-guided controller successfully resolves both quantity-based and difficulty-based optimization imbalances, driving the overall segmentation quality to state-of-the-art performance.

### 4.4. Small Lesion Experiment

The small lesion experiment focuses on disease regions that occupy only a small proportion of the leaf area. These samples are difficult because minor lesions can be suppressed during feature downsampling and may be confused with background noise, leaf veins, or natural texture variations. This experiment is designed to evaluate whether BFMambaNet can preserve sensitivity to small pathological regions while maintaining stable segmentation of larger leaf structures.

**Figure 7:**
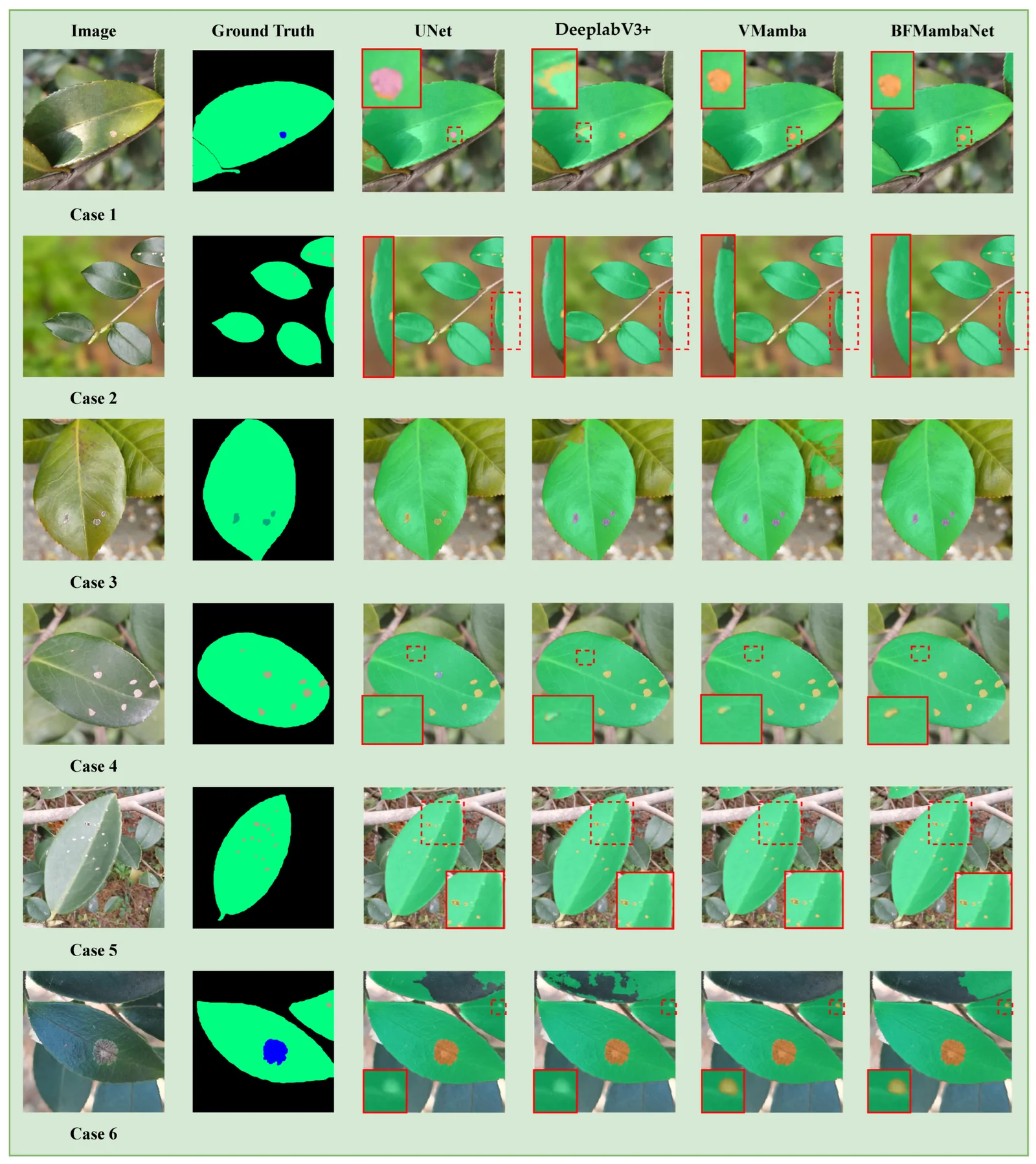
Qualitative comparison for small lesion segmentation. Red boxes indicate local regions where small pathological targets are difficult to detect.

Figure 7 shows the qualitative results of the small lesion experiment. The red boxes mark difficult local regions. In these regions, the disease targets occupy only a very small area, and their color or texture is close to the surrounding leaf surface. Therefore, the comparison focuses on whether each model can keep small lesion responses without producing obvious false positives around leaf veins, leaf edges, and background gaps.

As shown in Case 1, the lesion is a tiny spot on a high-contrast leaf surface. The highlighted region is easily confused with normal leaf texture. UNet and DeepLabV3+ produce enlarged or shifted responses in the red box, while VMamba weakens the local lesion boundary. In contrast, BFMambaNet keeps a compact response at the correct position. Its prediction is closer to the ground-truth mask in both location and shape.

In Case 2, the red boxes cover narrow leaf boundaries and small target regions near background gaps. This case is difficult because thin leaf edges and shadowed background structures may be misclassified as lesion pixels. The baseline models either lose part of the boundary structure or introduce extra responses around the edge. BFMambaNet preserves the main leaf contour and keeps the small lesion response more stable. This indicates that the proposed model can reduce edge-related false positives while retaining small disease details.

In Case 3, several small spots are distributed in the lower part of the leaf. These spots are sparse and discontinuous. The compared methods can detect part of the lesion signal, but their predictions are less consistent in shape and category color. BFMambaNet gives more continuous and cleaner small-region predictions. The red-box area shows that it separates the disease spots from the surrounding leaf texture more clearly.

Cases 4 and 5 contain dense dot-like lesions. The red boxes and enlarged regions show that many targets occupy only a few pixels. Such lesions are easily suppressed during repeated downsampling. UNet and DeepLabV3+ miss several small responses, and VMamba tends to smooth some local details. BFMambaNet preserves more discrete lesion dots and keeps their positions closer to the annotations. This result suggests that the wavelet-guided enhancement and boundary-frequency supervision are useful for recovering high-frequency lesion details.

In Case 6, the lesion appears in a region with dark leaf texture and background interference. The red-box area shows that some baseline outputs contain shifted or noisy responses. BFMambaNet produces a more localized prediction and suppresses irrelevant responses around the lesion. This demonstrates better robustness when small lesions are surrounded by confusing background patterns.

Overall, the small lesion experiment shows that BFMambaNet is more sensitive to weak and tiny pathological regions. More importantly, the model does not simply enlarge all suspicious responses. It keeps the predicted lesions compact and spatially aligned with the annotations. This behavior is important for plant disease segmentation, because small early-stage lesions often determine whether the model can provide reliable field-level diagnosis.

### 4.5. Coexistence Experiment

The coexistence experiment evaluates images containing multiple disease symptoms within the same leaf or image. These cases are challenging because different lesion categories may vary in scale, color, texture, and boundary clarity. The purpose of this experiment is to assess whether BFMambaNet can distinguish coexisting disease patterns and generate consistent segmentation masks under complex field conditions.

**Figure 8:**
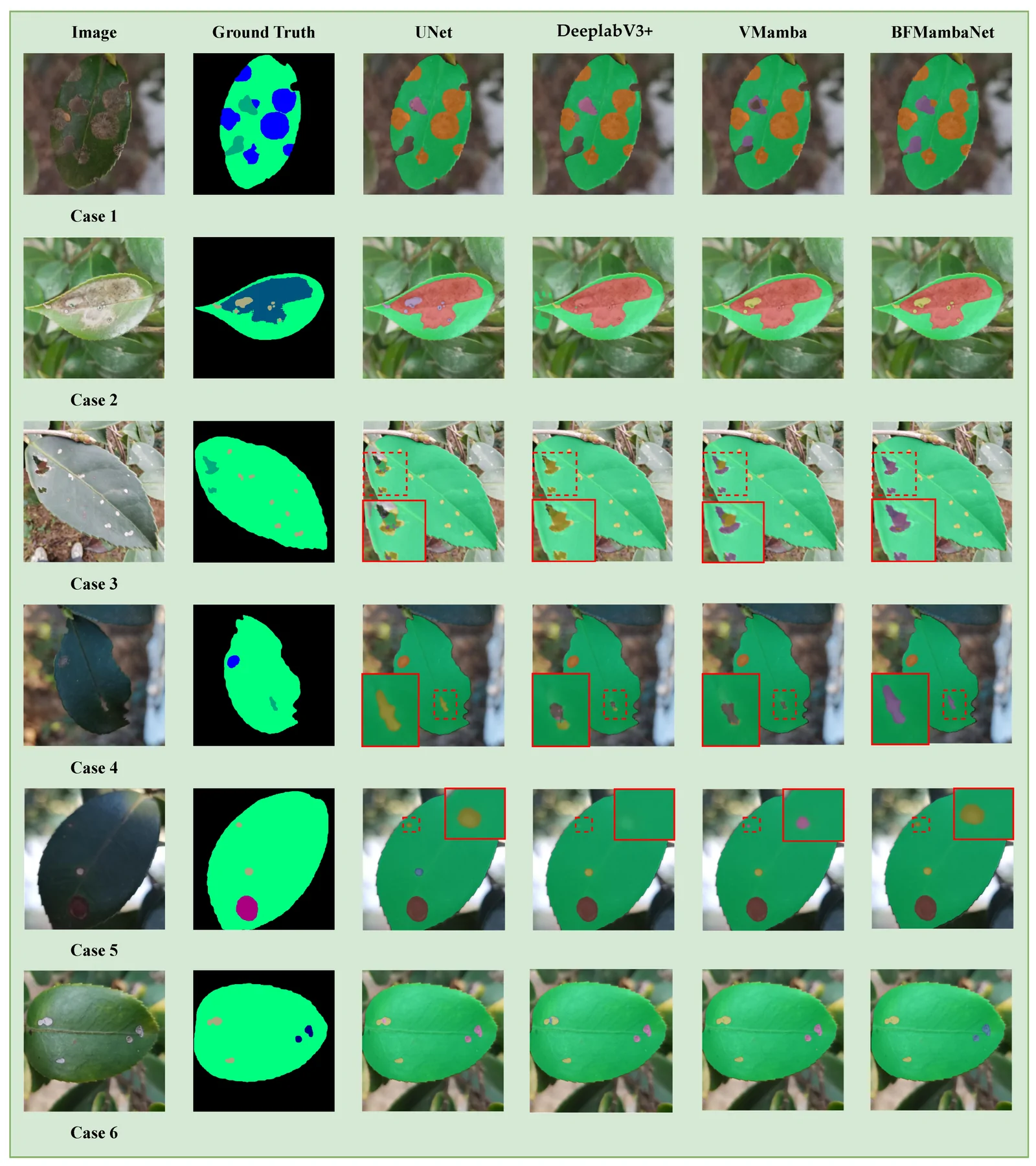
Qualitative comparison for coexistence segmentation. Red boxes indicate local regions where multiple disease patterns or small coexisting lesions are difficult to separate.

Figure 8 presents qualitative results for coexistence scenarios. Each row contains the original image, the ground-truth mask, and the predictions of UNet, DeepLabV3+, VMamba, and BFMambaNet. The red boxes mark local regions where different disease categories are close to each other, where small lesions appear beside large lesions, or where the lesion boundary is affected by shadows, leaf texture, and background interference.

In Case 1, several lesion categories appear on the same leaf and are distributed in separated regions. This case tests whether the model can maintain category consistency across scattered symptoms. UNet and DeepLabV3+ detect the main diseased areas, but some small regions are merged or assigned to inaccurate categories. VMamba gives a more stable leaf mask, but several local lesion boundaries are still less clear. BFMambaNet better separates the coexisting lesion regions and keeps their spatial distribution closer to the ground truth.

Case 2 contains a large irregular lesion together with small scattered spots. The large diseased area occupies a long region along the leaf surface, while the smaller targets are embedded near its boundary. This makes the prediction difficult because the model must segment both the dominant lesion and the minor coexisting symptoms. The baseline models tend to over-smooth the large lesion or miss small internal responses. BFMambaNet preserves the overall shape of the large lesion and also retains the small coexisting spots, which shows better balance between region-level segmentation and detail preservation.

In Case 3, the red boxes highlight a difficult region where two disease patterns appear close to the leaf edge. The enlarged areas show that the compared models can locate part of the diseased region, but they show category confusion between adjacent lesion types. UNet and DeepLabV3+ produce incomplete local predictions, and VMamba still mixes the neighboring categories in the highlighted area. BFMambaNet gives a clearer separation between the coexisting lesions and keeps the small dot-like regions on the leaf surface. This result suggests that the proposed global semantic modeling helps reduce confusion when different disease patterns are spatially close.

In Case 4, the red boxes focus on small coexisting lesions on a dark leaf surface. The low brightness and weak contrast make these regions easy to miss. The baseline models either produce shifted responses or confuse the small lesion with background texture. The dashed red box further shows that some small responses are unstable across the compared methods. BFMambaNet detects the small target more accurately and keeps the prediction more compact. It also avoids expanding the lesion into the surrounding healthy leaf area.

Case 5 contains a clear large lesion and a very small lesion near the upper leaf region, as marked by the red boxes. This is a typical coexistence case with strong scale variation. The lower lesion is relatively easy to detect, but the upper small spot is weak and can be suppressed during feature extraction. UNet and DeepLabV3+ miss or weaken this small spot, and VMamba predicts it with less reliable category information. BFMambaNet preserves both the lower large lesion and the upper small lesion. The prediction is closer to the ground truth in position and category consistency.

In Case 6, multiple small lesions are distributed on a clean leaf surface. Although the background is less complex, the disease regions are small and belong to different categories. The compared models can detect most visible spots, but their category assignment is less stable, especially for the small lesions near the right side of the leaf. BFMambaNet produces more consistent small-region predictions and better separates the coexisting categories. This indicates that the proposed method is not only effective for large mixed lesions but also useful for sparse coexistence patterns.

Overall, the coexistence experiment shows that BFMambaNet can distinguish multiple disease patterns within the same image more reliably than the compared models. The improvement is especially clear in the red-box regions, where lesion categories are close, lesion scales differ greatly, or the local contrast is weak. These results demonstrate that the combination of global semantic modeling, wavelet-guided enhancement, and boundary-frequency supervision improves category discrimination and structural consistency in complex coexistence scenarios.

### 4.6. Efficiency Evaluation

A quantitative cost analysis was conducted to assess the computational overhead introduced by BFMambaNet relative to the baseline U-Mamba. Three key metrics were measured: model parameter count, floating-point operations (FLOPs), and inference throughput (FPS). The results are presented in Table 10.

**Table 10:** Cost Analysis Calculation.

Method	Params	FLOPs	FPS
U-Mamba	64.8 M	57 G	41
BFMambaNet (Ours)	86.9 M	65 G	30

As shown in Table 10, introducing the multi-branch boundary-frequency design raises the trainable parameter count from 64.8 M to 86.9 M—a relative increase of 34%—accompanied by a proportionally modest growth in FLOPs from 57 G to 65 G (approximately 14%). The expanded model capacity directly supports richer multi-scale feature extraction and more precise lesion boundary delineation. Correspondingly, inference throughput decreases from 41 FPS to 30 FPS, reflecting the inherent cost-accuracy trade-off associated with a more expressive network architecture.

At 30 FPS on GPU-enabled hardware, BFMambaNet sustains near-real-time throughput sufficient for practical deployment in GPU-accelerated agricultural inspection pipelines, cloud-based plant health monitoring services, and laboratory research workstations. Deployment on severely resource-constrained devices without GPU support remains a challenge given the current model size, which we treat as an acknowledged limitation rather than an intended application scenario.

To improve deployability in resource-limited settings, future efforts will focus on two complementary compression strategies: (1) structured pruning targeting redundant channels and layers identified through sensitivity analysis; and (2) knowledge distillation, where a lightweight student network is guided by BFMambaNet to retain competitive segmentation quality at significantly reduced computational cost.

### 4.7. Generalization Experiment

To evaluate the generalization ability, robustness, and transferability of BFMambaNet across different crop species and imaging environments, we conducted cross-dataset validation on an external Apple leaf disease dataset [65]. In real-world agronomic deployment, disease segmentation models are often required to operate in heterogeneous environments, facing unseen leaf patterns, varying color profiles, and different illumination conditions. Testing the model on a completely independent crop species provides a rigorous assessment of whether the architectural features learned by our framework are crop-specific or generally applicable to plant leaf pathology segmentation.

**Figure 9:**
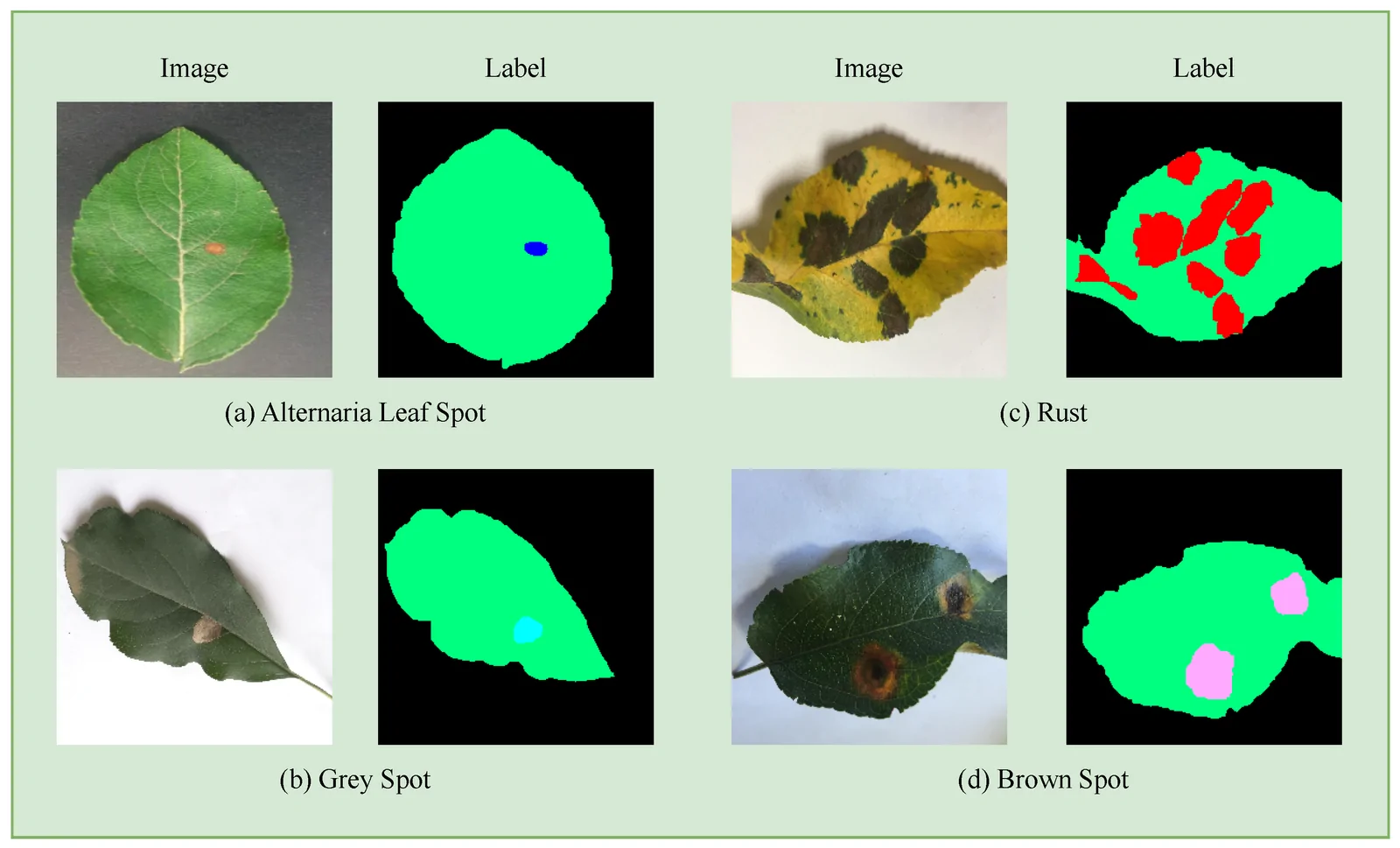
Representative disease categories in the apple leaf disease generalization dataset. (**a**) Alternaria Leaf Spot, (**b**) Grey Spot, (**c**) Rust, and (**d**) Brown Spot.

The curated Apple disease dataset serves as our external validation benchmark, containing 1000 leaf images across four typical disease categories: Alternaria Leaf Spot, Grey Spot, Rust, and Brown Spot. These diseases present distinct visual features, including concentric ring patterns, tiny discrete rust spots, and fuzzy lesion boundaries, as illustrated in Figure 9. In this experiment, all models were trained and tested on the Apple dataset under the same configuration to evaluate their adaptability to the new target domain.

We benchmarked BFMambaNet against U-Mamba [47] under identical experimental conditions across both the original *Camellia oleifera* dataset and the external Apple generalization dataset. The quantitative performance evaluation and comparison are summarized in Table 11. The experimental results demonstrate that BFMambaNet consistently outperforms U-Mamba on both datasets across all metrics. Specifically, on the *Camellia oleifera* dataset, BFMambaNet achieves an mIoU of 85.46% and a Dice coefficient of 91.26%, yielding an improvement of 3.73% and 3.23% over U-Mamba, respectively. On the Apple leaf disease dataset, our model obtains an mIoU of 82.91% and a Dice coefficient of 90.67%, outperforming U-Mamba by 3.39% and 3.84%. This consistent improvement demonstrates that BFMambaNet’s design generalizes effectively to unseen crop disease segmentation tasks without overfitting to the specific leaf structures of the training set.

**Figure 10:**
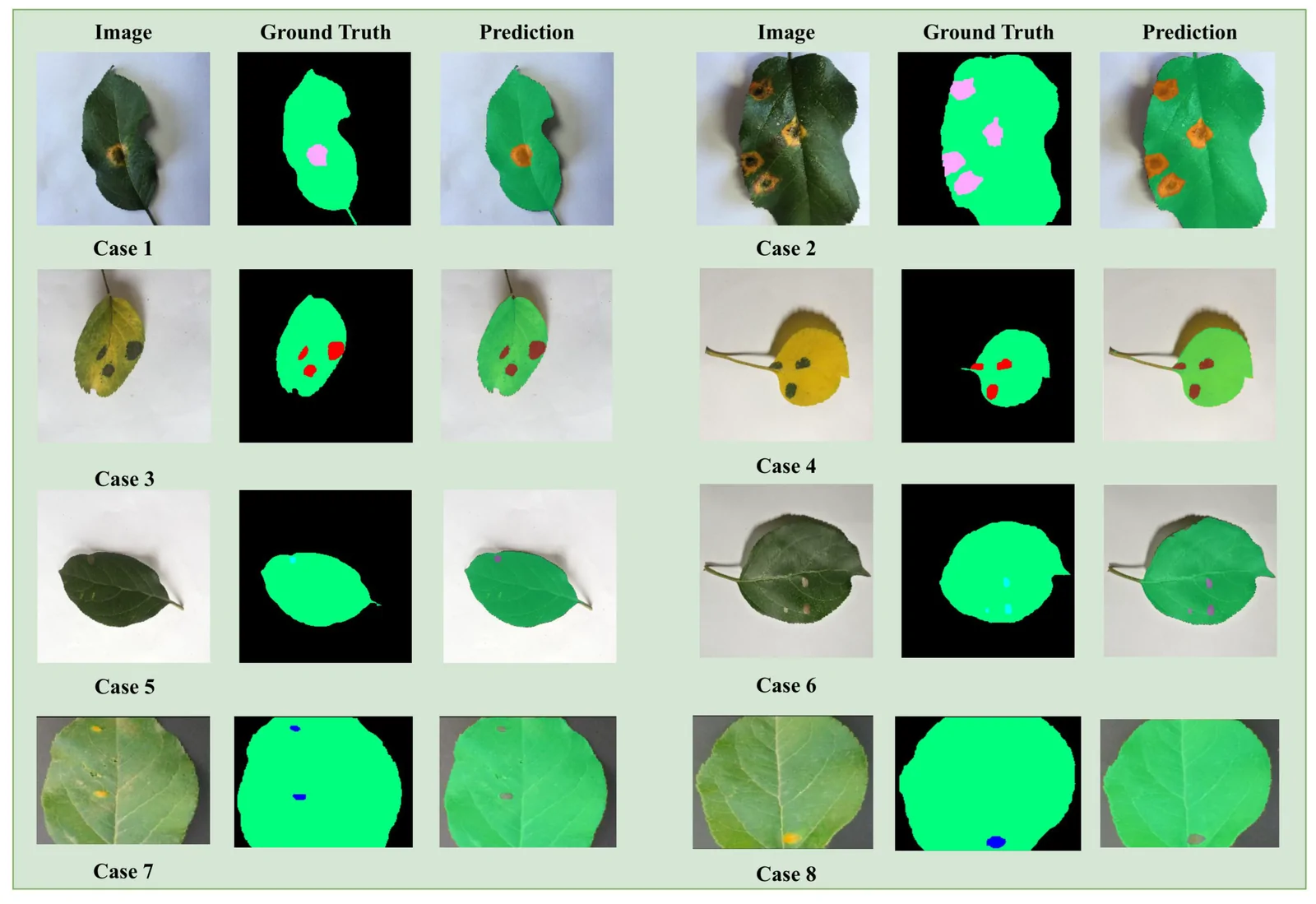
Qualitative segmentation results of BFMambaNet on the Apple leaf disease generalization dataset. Each row shows the original image, ground-truth mask, and BFMambaNet prediction for representative disease categories.

The superior performance of BFMambaNet on the unseen Apple leaf disease dataset is attributed to its semantic-boundary-frequency multi-branch design. As illustrated in Figure 10, BFMambaNet consistently generates accurate and complete segmentation masks across all four disease categories. The SSFM block successfully models the long-range semantic dependencies of scattered spot patterns on apple leaves, helping to distinguish true pathological regions from complex background clutter. Concurrently, the GWSE block effectively preserves the local high-frequency boundary cues, ensuring that the model remains highly sensitive to tiny lesions (such as Rust spots) and irregular contours. Furthermore, the RL-guided adaptive loss controller dynamically adjusts the multi-objective optimization (region consistency, boundary accuracy, and frequency details) on the new dataset, preventing the optimization process from being dominated by simple classes.

We also note a slight performance decrease compared to the Camellia dataset. This is a typical consequence of domain shift, resulting from leaf structure differences, distinct color distributions, and variations in imaging illumination or background noise. Nevertheless, the fact that BFMambaNet maintains a stable performance advantage over the baseline U-Mamba validates its cross-species generalization capability. This demonstrates that our proposed model possesses high robustness and can be applied as a general tool for multiple crop leaf disease diagnostics.

**Table 11:** Cross-dataset performance evaluation and comparison of the segmentation models on the *Camellia oleifera* and Apple leaf disease datasets. The highest value is highlighted in bold.

Dataset	Method	mIoU (%)	Precision (%)	Recall (%)	Dice (%)
* Camellia oleifera *	U-Mamba	81.73 ± 0.31	89.12 ± 0.38	87.05 ± 0.45	88.03 ± 0.38
	BFMambaNet (Ours)	** 85.46 ± 0.23 **	** 92.39 ± 0.48 **	** 91.43 ± 0.24 **	** 91.26 ± 0.28 **
Apple	U-Mamba	79.52 ± 0.34	87.12 ± 0.38	86.54 ± 0.45	86.83 ± 0.29
	BFMambaNet (Ours)	** 82.91 ± 0.30 **	** 91.24 ± 0.42 **	** 90.12 ± 0.26 **	** 90.67 ± 0.31 **

### 4.8. Sensitivity Analysis

To verify the stability and robustness of BFMambaNet under different configurations, we conducted parameter sensitivity experiments focusing on: (1) the injection depth of the GWSE blocks, (2) the hyperparameters of the RL-guided adaptive loss controller, and (3) the kernel size of the boundary supervision extraction. All sensitivity results were evaluated using 5-fold cross-validation, and the default settings are highlighted in bold. The quantitative evaluation is summarized in Table 12.

**Table 12:** Parameter sensitivity analysis of GWSE stages, RL update interval $I_{\mathrm{update}}$, momentum smoothing factor α, warm-up epochs $E_{\mathrm{warmup}}$, and boundary extraction kernel size k×k on BFMambaNet. The default settings are highlighted in bold.

Parameter Type	Configuration	Dice (%)	mIoU (%)	Boundary IoU (%)	NSD (%)
GWSE Stages	0 stage (w/o GWSE)	88.75 ± 0.27	83.67 ± 0.20	78.92 ± 0.38	80.95 ± 0.34
	1 stage	90.85 ± 0.32	84.95 ± 0.41	82.68 ± 0.40	85.12 ± 0.39
	** 3 stages **	** 91.82 ± 0.35 **	** 85.88 ± 0.30 **	** 84.08 ± 0.29 **	** 86.52 ± 0.31 **
	All stages (Default)	91.26 ± 0.28	85.46 ± 0.23	83.91 ± 0.35	86.36 ± 0.34
Update Interval $I_{\mathrm{update}}$	$I_{\mathrm{update}}=1$	89.92 ± 0.72	83.85 ± 0.68	82.02 ± 0.75	84.11 ± 0.69
	$I_{\mathrm{update}}=5$ **(Default)**	** 91.26 ± 0.28 **	** 85.46 ± 0.23 **	** 83.91 ± 0.35 **	** 86.36 ± 0.34 **
	$I_{\mathrm{update}}=10$	90.95 ± 0.41	85.12 ± 0.43	83.21 ± 0.48	85.80 ± 0.42
	$I_{\mathrm{update}}=20$	90.58 ± 0.25	84.84 ± 0.32	82.52 ± 0.30	85.11 ± 0.29
Smoothing Factor α	α=0.1	90.84 ± 0.35	85.02 ± 0.38	82.95 ± 0.42	85.61 ± 0.39
	α=0.3 **(Default)**	** 91.26 ± 0.28 **	** 85.46 ± 0.23 **	** 83.91 ± 0.35 **	** 86.36 ± 0.34 **
	α=0.5	91.03 ± 0.42	85.34 ± 0.46	83.42 ± 0.48	85.95 ± 0.45
	α=0.8	89.15 ± 0.88	83.21 ± 0.95	80.52 ± 1.02	82.44 ± 0.98
Warm-up Epochs $E_{\mathrm{warmup}}$	$E_{\mathrm{warmup}}=10$	90.72 ± 0.39	84.92 ± 0.42	82.90 ± 0.45	85.38 ± 0.41
	$E_{\mathrm{warmup}}=20$ **(Default)**	** 91.26 ± 0.28 **	** 85.46 ± 0.23 **	** 83.91 ± 0.35 **	** 86.36 ± 0.34 **
	$E_{\mathrm{warmup}}=50$	90.64 ± 0.33	84.88 ± 0.36	82.81 ± 0.40	85.22 ± 0.35
Boundary Kernel Size *k*	k=3 **(Default)**	** 91.26 ± 0.28 **	** 85.46 ± 0.23 **	** 83.91 ± 0.35 **	** 86.36 ± 0.34 **
	k=5	91.15 ± 0.38	84.98 ± 0.42	81.52 ± 0.40	83.88 ± 0.39
	k=7	90.68 ± 0.43	84.22 ± 0.48	77.10 ± 0.52	79.45 ± 0.50

*GWSE Block Layers Sensitivity*: We analyzed the injection depth of high-frequency spatial gate by configuring the resolution stages at which GWSE is active. The default configuration of BFMambaNet incorporates GWSE blocks at all stages (up to 512 resolution), achieving a baseline SOTA performance of 91.26% Dice and 85.46% mIoU. When the GWSE block is completely deactivated (0 stages), the performance drops to a lower level of 88.75% Dice and 83.67% mIoU, demonstrating that without wavelet-guided high-frequency supervision, the network is prone to boundary blurring and misclassifying leaf veins as pathological regions. Interestingly, when we restrict the GWSE blocks to the deep feature stages (resolutions from 8 to 64, or 1 to 3 stages), the performance increases to 91.82% Dice, 85.88% mIoU, 84.08% Boundary IoU, and 86.52% NSD, which is better than the original default “all stages” configuration. However, since the “all stages” configuration was established *a priori* based on the theoretical design of capturing global multi-scale features, it was retained as the default baseline for the experiments to ensure rigorous independent evaluation and prevent circular validation bias. Physically, deep feature maps carry high abstract semantic capacity, and the 2D DWT efficiently filters out low-frequency redundant semantics to capture narrow boundaries. However, when extending GWSE to all stages (including shallow layers at 256 and 512 resolutions), the high-resolution layers introduce severe physical noise (e.g., healthy leaf edges, veins, or background soil textures) that is incorrectly amplified as pathological boundaries. This leads to a substantial increase in false-positive predictions, thereby dragging down the Precision and degrading the final Dice and mIoU compared to the optimal 3-stage setup.

*RL Controller Hyperparameters Sensitivity*: We evaluated three key parameters governing the stabilization, exploration, and convergence of the adaptive loss meta-controller. First, for the update interval $I_{\mathrm{update}}$, updating the weights at every epoch ($I_{\mathrm{update}}=1$) results in severe training oscillations, low performance (89.92% Dice), and high variance. The minor validation fluctuations are over-interpreted by the RL agent, resulting in chaotic gradient updates. Setting $I_{\mathrm{update}}=5$ achieves the highest and most stable performance, as 5 epochs allow the segmentation network to digest the updated weights and output high-quality performance metrics. Slow updates ($I_{\mathrm{update}}=20$) lead to performance decay, approaching the fixed-weight baseline since the agent fails to track the fast training dynamics. Second, the momentum smoothing factor α controls weight transition steps. A conservative setting (α=0.1) slows down error correction, yielding suboptimal early convergence. The default setting (α=0.3) balances adaptability and smoothness. When α≥0.8, the loss curves exhibit jagged fluctuations and potential training instability due to massive gradient steps caused by sudden weight jumps. Third, the warm-up epochs $E_{\mathrm{warmup}}$ determine when RL control begins. A premature start ($E_{\mathrm{warmup}}=10$) traps the model in local saddle points, as the early chaotic representations mislead the policy network. The default ($E_{\mathrm{warmup}}=20$) allows the backbone to establish basic representations of leaf and background structures before targeting complex disease categories. Delaying the start to $E_{\mathrm{warmup}}=50$ retards training convergence and allows easy categories (such as Soft Rot) to dominate the early optimization, suppressing the gradient acquisition of difficult classes in later stages.

*Dilation/Erosion Kernel Size Sensitivity*: This experiment defines the width of the pseudo-boundary bands used for boundary auxiliary supervision. The default 3×3 kernel yields a tight boundary band, forcing the network to focus on high-frequency details within the exact contour transition zone, yielding the highest Boundary IoU (83.91%) and NSD (86.36%). A 5×5 kernel provides a wider margin that accommodates annotation errors but slightly blurs micro-scale details. A wider 7×7 kernel degenerates the boundary supervision line into a broad band, permitting larger spatial deviations and leading to rounded or wider predicted boundaries, which severely degrades Boundary IoU (77.10%) and NSD (79.45%).

### 4.9. Robustness Evaluation on Challenging Subsets

To further verify the generalization and robustness of BFMambaNet under practical field conditions, we partitioned the test set into three challenging subsets based on typical environmental factors: (1) *Uneven Illumination (UI)*: 47 images containing strong specular highlights, dense foliage shadows, or low-contrast ambient light; (2) *Complex Background (CB)*: 87 images with complex orchard clutter, soil, weeds, or branches in the background; and (3) *Coexisting Symptoms (CS)*: 36 images where multiple disease symptoms occur concurrently on a single leaf. These subsets represent the natural occurrence rates of the corresponding environmental challenges within our independent test set (consisting of 140 images), where 33.57%, 62.14%, and 25.71% of the test images exhibit uneven illumination, complex backgrounds, and coexisting symptoms, respectively. We compared BFMambaNet with three representative baselines: UNet (convolutional baseline), DeepLabV3+ (context-aggregation baseline), and VMamba (state-space baseline). The quantitative results are presented in Table 13.

As shown in the table, all models experience performance degradation compared to the overall test results, confirming that these filtered subsets represent highly difficult segmentation scenarios. However, BFMambaNet exhibits significantly higher resistance to these factors. Under uneven illumination (UI), BFMambaNet outperforms the state-of-the-art VMamba by 3.70% mIoU. This is because the GWSE block processes feature maps in the wavelet frequency domain, extracting illumination-invariant high-frequency gradients. Under complex backgrounds (CB), our model maintains an mIoU of 82.15% (outperforming VMamba by 3.55%), proving that the global semantic modeling in the SSFM block successfully filters out distracting orchard backgrounds. Most notably, for the coexisting symptoms (CS) subset, which is highly challenging due to optimization competition among multiple disease classes, BFMambaNet leads VMamba by a massive margin of 5.10% mIoU (81.30% vs. 76.20%). This substantial advantage directly validates that the RL-guided adaptive loss controller effectively balances the learning trajectories of multiple competing classes, preventing minority or harder symptoms from being neglected during joint training.

## 5. Conclusions

This study aims to address the fine-grained segmentation problem of *Camellia oleifera* leaf diseases under complex field conditions. In practical orchard scenes, disease regions often show weak boundaries, small lesion areas, uneven illumination, background interference, and coexisting symptoms from different categories. These factors make pixel-level disease segmentation more difficult than general leaf region extraction. To solve these problems, we proposed BFMambaNet, a Boundary-Frequency-guided Global Semantic Mamba Network. The model combines global semantic Mamba modeling, gated wavelet spatial enhancement, boundary-frequency auxiliary supervision, and reinforcement-learning-guided adaptive loss control within a unified encoder-decoder framework.

Experimental results demonstrate that BFMambaNet achieves strong and stable segmentation performance. Compared with representative CNN-based, Transformer-based, and Mamba-based segmentation models, the proposed method obtains the best overall results, with a Precision of 92.39%, Recall of 91.43%, Dice of 91.26%, and mIoU of 85.46%. Compared with VMamba, BFMambaNet improves mIoU from 83.42% to 85.46%. The class-wise IoU results further show that the proposed method performs well on difficult disease categories such as Anthracnose, Worm Holes, Red Spot, Soft Rot, and Algae Spot. These categories usually contain irregular lesion shapes, low-contrast boundaries, or visual patterns similar to healthy leaf textures. Therefore, the improvement indicates that BFMambaNet can better distinguish subtle pathological regions from complex field backgrounds. In addition, quantitative evaluations on challenging test subsets demonstrate that BFMambaNet achieves superior robustness under extreme field conditions, outperforming the SOTA VMamba by margins of 3.70% mIoU under uneven illumination (UI), 3.55% mIoU under complex backgrounds (CB), and 5.10% mIoU under coexisting symptoms (CS). Furthermore, cross-dataset validation on an external Apple leaf disease dataset demonstrates the model’s high generalization capability, outperforming the baseline U-Mamba by 3.39% mIoU and 3.84% Dice. Computational cost analysis indicates that BFMambaNet achieves a competitive inference speed of 30 FPS under sliding-window settings, representing a practical trade-off between segmentation accuracy and resource requirements.

The ablation experiments further verify the effectiveness of each component. The SSFM block enhances long-range semantic dependency modeling and helps connect scattered lesion regions. The GWSE block improves boundary-sensitive feature refinement by introducing wavelet-domain high-frequency cues. The internal ablation of GWSE also shows that the spatial branch, local value branch, frequency fusion, and learnable gate provide complementary effects. Boundary-frequency auxiliary supervision strengthens contour localization and fine-detail recovery during training. In addition, the RL-guided adaptive loss controller improves optimization stability by adjusting class-wise reweighting factors and the weights of segmentation, boundary, and frequency losses according to the training state. The qualitative comparison, small lesion experiment, coexistence experiment, and robust subset filtering analysis also confirm that BFMambaNet produces more complete lesion masks, clearer boundaries, and more reliable category predictions in difficult visual scenarios. Moreover, systematic analysis of the training dynamics and loss curves demonstrates that BFMambaNet achieves faster convergence and a smoother validation trajectory than U-Mamba. Statistical distribution of the learnable parameters validates that the RL controller successfully resolves severe class-imbalance by dynamically allocating higher penalties to underrepresented categories (e.g., Anthracnose) while compressing the gradient contributions of easy-to-segment symptoms (e.g., Soft Rot) as training progresses.

In summary, BFMambaNet provides an effective pixel-level segmentation framework for *Camellia oleifera* leaf disease analysis. It can support downstream agricultural applications such as disease severity assessment, field disease monitoring, and intelligent orchard management. Nevertheless, this study still has some limitations. The current framework mainly relies on RGB images, and the dataset size is still limited compared with large-scale agricultural vision benchmarks. In future work, we will extend BFMambaNet toward a multimodal disease segmentation framework. Textual symptom descriptions, environmental information, or other sensing modalities can be introduced as auxiliary semantic priors to further reduce visual ambiguity. We will also explore larger multi-region datasets, domain adaptation strategies, and lightweight model designs to improve generalization ability and practical deployment in real field environments. In particular, we acknowledge the current limitation in validating BFMambaNet on large-scale benchmarks, which is primarily constrained by the available GPU resources in our laboratory. As our computational infrastructure improves, we commit to prioritizing comprehensive evaluations on large-scale plant disease datasets as a key direction of our ongoing research.

## Figures and Tables

**Table 1:** Number of images containing each disease category. The proportions are calculated using the total number of images (1400) as the explicit denominator. Because some images contain multiple coexisting disease categories, the sum of all proportions naturally exceeds 100%.

Disease Category	Quantity	Proportion
Tea Sooty Mold	219	15.6%
Tea White Scab	554	39.6%
Algae Leaf Spot	203	14.5%
Worm Holes	238	17.0%
Anthracnose	134	9.6%
Red Leaf Spot	200	14.3%
Soft Rot	203	14.5%
Total number of images	1400	-

**Table 2:** Detailed network architecture of BFMambaNet. The network consists of 8 stages. The table maps the specific module compositions (Residual Block, SSFM Block, and GWSE Block) corresponding to the conceptual diagram in Figure 3.

Stage	Resolution	Encoder		Decoder		Skip Connection
		Channels	Components	Channels	Components	
Input	512×512	3	-	-	-	-
Stage 1	512×512	32	2 × Residual	32	2 × Residual, 1 × GWSE	Concat.
Stage 2	256×256	64	2 × Residual, 1 × SSFM	64	2 × Residual, 1 × GWSE	Concat.
Stage 3	128×128	128	2 × Residual, 1 × SSFM	128	2 × Residual, 1 × GWSE	Concat.
Stage 4	64×64	256	2 × Residual, 1 × SSFM	256	2 × Residual, 1 × GWSE	Concat.
Stage 5	32×32	512	2 × Residual, 1 × SSFM	512	2 × Residual, 1 × GWSE	Concat.
Stage 6	16×16	512	2 × Residual, 1 × SSFM	512	2 × Residual, 1 × GWSE	Concat.
Stage 7	8×8	512	2 × Residual, 1 × SSFM	512	2 × Residual, 1 × GWSE	Concat.
Stage 8 (Bottleneck)	4×4	512	2 × Residual, 1 × SSFM	-	-	-
** Downsampling (Encoder): ** Strided Convolution (3×3, Stride = 2)						
** Upsampling (Decoder): ** Nearest Neighbor Interpolation + 1×1 Convolution						
** Residual Block Config: ** Conv (3×3) → Instance Norm → LeakyReLU						

**Table 3:** Hardware Devices and Software Environment.

Environment	Device	Parameter
Hardware environment	CPU	Intel(R) Xeon(R) Platinum 8481C
	GPU	NVIDIA GeForce RTX 4090
	RAM	64 G
	Video memory	24 G
Software environment	OS	Ubuntu20.04
	CUDA Toolkit	11.8
	Python	3.8
	Pytorch-GPU	2.0.0
	torchvision	0.16.1

**Table 4:** Model Training Hyperparameters.

Hyperparameters	Parameter
Image Size	512 × 512
Batch Size	4
Initial learning rate	0.0002
Optimizer	AdamW

**Table 13:** Robustness comparison (Dice and mIoU) under three challenging subsets: Uneven Illumination (UI), Complex Background (CB), and Coexisting Symptoms (CS). The highest value is highlighted in bold.

Methods	UI (47 Images)		CB (87 Images)		CS (36 Images)	
	Dice (%)	mIoU (%)	Dice (%)	mIoU (%)	Dice (%)	mIoU (%)
UNet	83.10 ± 0.52	74.20 ± 0.48	81.90 ± 0.58	72.85 ± 0.54	80.50 ± 0.65	71.10 ± 0.61
DeepLabV3+	85.12 ± 0.44	76.50 ± 0.40	84.20 ± 0.49	75.40 ± 0.44	82.80 ± 0.50	73.90 ± 0.48
VMamba	87.24 ± 0.38	79.10 ± 0.35	86.80 ± 0.42	78.60 ± 0.39	84.90 ± 0.45	76.20 ± 0.42
** BFMambaNet (Ours) **	** 90.05 ± 0.28 **	** 82.80 ± 0.24 **	** 89.65 ± 0.30 **	** 82.15 ± 0.26 **	** 89.10 ± 0.34 **	** 81.30 ± 0.31 **

## Data Availability

Requests to access the datasets and the code should be sent via email to hao20233494@163.com.

## References

[1] Quan W., Wang A., Gao C., Li C. (2022). Applications of Chinese *Camellia oleifera* and its by-products: A review. Front. Chem..

[2] Ma J., Ye H., Rui Y., Chen G., Zhang N. (2010). Fatty acid composition of *Camellia oleifera* oil. J. Verbrauch. Lebensm..

[3] Zhang S., Li X., Li C., Chen X. (2017). Anti-inflammatory and antioxidative effects of *Camellia oleifera* Abel components. Future Med. Chem..

[4] Yang C., Liu X., Chen Z., Lin Y., Wang S. (2016). Comparison of oil content and fatty acid profile of ten new *Camellia oleifera* cultivars. J. Lipids.

[5] Zhou A., Wang F., Yin J., Peng R., Deng J., Shen D., Wu J., Liu X., Ma H. (2022). Antifungal action and induction of resistance by *Bacillus* sp. strain YYC 155 against *Colletotrichum fructicola* for control of anthracnose disease in *Camellia oleifera*. Front. Microbiol..

[6] Zhou A., Zhou H., Peng R., Liu D., Wu J., Deng J., Wang F. (2025). Melatonin-induced *Bacillus tequilensis* enhanced the disease resistance of *Camellia oleifera* against anthracnose by modulating cell wall and phenylpropanoid metabolism. Front. Plant Sci..

[7] Strange R.N., Scott P.R. (2005). Plant disease: A threat to global food security. Annu. Rev. Phytopathol..

[8] Sankaran S., Mishra A., Ehsani R., Davis C. (2010). A review of advanced techniques for detecting plant diseases. Comput. Electron. Agric..

[9] Dolatabadian A., Neik T.X., Danilevicz M.F., Upadhyaya S.R., Batley J., Edwards D. (2025). Image-based crop disease detection using machine learning. Plant Pathol..

[10] Singh V., Misra A.K. (2017). Detection of plant leaf diseases using image segmentation and soft computing techniques. Inf. Process. Agric..

[11] Hegde R.B., Prasad K., Hebbar H., Singh B.M.K. (2019). Comparison of traditional image processing and deep learning approaches for classification of white blood cells in peripheral blood smear images. Biocybern. Biomed. Eng..

[12] Rosca C.M. (2023). Comparative Analysis of Object Classification Algorithms: Traditional Image Processing Versus Artificial Intelligence—Based Approach. Rom. J. Pet. Gas Technol..

[13] Marias K. (2021). The constantly evolving role of medical image processing in oncology: From traditional medical image processing to imaging biomarkers and radiomics. J. Imaging.

[14] Mohanty S.P., Hughes D.P., Salathé M. (2016). Using deep learning for image-based plant disease detection. Front. Plant Sci..

[15] Shoaib M., Hussain T., Shah B., Ullah I., Shah S.M., Ali F., Park S.H. (2022). Deep learning-based segmentation and classification of leaf images for detection of tomato plant disease. Front. Plant Sci..

[16] Kayalibay B., Jensen G., van der Smagt P. (2017). CNN-based segmentation of medical imaging data. arXiv.

[17] Gidaris S., Komodakis N. Object detection via a multi-region and semantic segmentation-aware CNN model. Proceedings of the IEEE International Conference on Computer Vision.

[18] Xu K., Wen L., Li G., Bo L., Huang Q. Spatiotemporal CNN for video object segmentation. Proceedings of the IEEE/CVF Conference on Computer Vision and Pattern Recognition.

[19] Wrat G., Ranjan P., Mishra S.K., Jose J.T., Das J. (2024). Neural network-enhanced internal leakage analysis for efficient fault detection in heavy machinery hydraulic actuator cylinders. Proc. Inst. Mech. Eng. Part C J. Mech. Eng. Sci..

[20] Chen L.C., Papandreou G., Kokkinos I., Murphy K., Yuille A.L. (2018). DeepLab: Semantic image segmentation with deep convolutional nets, atrous convolution, and fully connected CRFs. IEEE Trans. Pattern Anal. Mach. Intell..

[21] Zhou J., Hao M., Zhang D., Zou P., Zhang W. (2019). Fusion PSPnet image segmentation based method for multi-focus image fusion. IEEE Photonics J..

[22] Yu C., Xiao B., Gao C., Yuan L., Zhang L., Sang N., Wang J. Lite-HRNet: A lightweight high-resolution network. Proceedings of the IEEE/CVF Conference on Computer Vision and Pattern Recognition.

[23] Vaswani A., Shazeer N., Parmar N., Uszkoreit J., Jones L., Gomez A.N., Kaiser L., Polosukhin I. (2017). Attention is all you need. Advances in Neural Information Processing Systems.

[24] Han K., Xiao A., Wu E., Guo J., Xu C., Wang Y. (2021). Transformer in transformer. Advances in Neural Information Processing Systems.

[25] Liu Y., Tian Y., Zhao Y., Yu H., Xie L., Wang Y., Ye Q., Jiao J., Liu Y. (2024). VMamba: Visual state space model. Advances in Neural Information Processing Systems.

[26] Lu P., Huang J., Zeng Q., Wang X., Chen B., Langlais P., Cui Y., Belgrave D., Zhang C., Lin H., Pascanu R., Koniusz P., Ghassemi M., Chen N. (2025). Mamba Modulation: On the Length Generalization of Mamba Models. Advances in Neural Information Processing Systems.

[27] Zhang Y., Li X., Wang J., Chen Y. (2026). Vision Mamba in remote sensing: A comprehensive survey of techniques, applications and outlook. Remote Sens..

[28] Choi H., Baraniuk R.G. (2001). Multiscale image segmentation using wavelet-domain hidden Markov models. IEEE Trans. Image Process..

[29] Anusooya G., Bharathiraja S., Mahdal M., Sathyarajasekaran K., Elangovan M. (2023). Self-supervised wavelet-based attention network for semantic segmentation of MRI brain tumor. Sensors.

[30] Tian Z., Si X., Zheng Y., Chen Z., Li X. (2020). Multi-step medical image segmentation based on reinforcement learning. J. Ambient. Intell. Humaniz. Comput..

[31] Allioui H., Mohammed M.A., Benameur N., Al-Khateeb B., Abdulkareem K.H., Garcia-Zapirain B., Damaševičius R., Maskeliūnas R. (2022). A multi-agent deep reinforcement learning approach for enhancement of COVID-19 CT image segmentation. J. Pers. Med..

[32] Hou Q., Cheng M.M., Hu X., Borji A., Tu Z., Torr P. Deeply supervised salient object detection with short connections. Proceedings of the IEEE Conference on Computer Vision and Pattern Recognition.

[33] Sun D., Yao A., Zhou A., Zhao H. Deeply-supervised knowledge synergy. Proceedings of the IEEE/CVF Conference on Computer Vision and Pattern Recognition.

[34] Kervadec H., Bouchtiba J., Desrosiers C., Granger E., Dolz J., Ben Ayed I. Boundary loss for highly unbalanced segmentation. Proceedings of the 2nd International Conference on Medical Imaging with Deep Learning.

[35] Borse S., Wang Y., Zhang Y., Porikli F. InverseForm: A loss function for structured boundary-aware segmentation. Proceedings of the IEEE/CVF Conference on Computer Vision and Pattern Recognition.

[36] Lu J., Tan L., Jiang H. (2021). Review on convolutional neural network (CNN) applied to plant leaf disease classification. Agriculture.

[37] Lee S.H., McDowell S., Leslie C., McCreery K., Earles M., Brown P.J. (2025). Comparison of Mask-R-CNN and thresholding-based segmentation for high-throughput phenotyping of walnut kernel color. Plants.

[38] Liu Z., Feng Q., Qin C., Lin Y., Xia P., Wang H., Gong L., Liu C. (2026). EDSC-HRAFNet: An apple tree branch semantic segmentation model for harvesting robots under complex orchard conditions. Artif. Intell. Agric..

[39] Zheng H., Li D., Xu X., Quan L., Zhu Y., Xiao M. (2026). Unsupervised geometry-driven DSM terrain correction for Rice seedling segmentation in flooded Paddy fields. Artif. Intell. Agric..

[40] Kitzler F., Bauer A., Kruder-Motsch V. (2026). Beyond Color: Advanced RGB-D data augmentation for robust semantic segmentation in crop farming scenes. Comput. Electron. Agric..

[41] Xie E., Wang W., Yu Z., Anandkumar A., Alvarez J.M., Luo P. (2021). SegFormer: Simple and efficient design for semantic segmentation with transformers. Advances in Neural Information Processing Systems.

[42] Cheng B., Choudhuri A., Misra I., Kirillov A., Girdhar R., Schwing A.G. (2021). Mask2Former for video instance segmentation. arXiv.

[43] Ravi N., Gabeur V., Hu Y.T., Hu R., Ryali C., Ma T., Khedr H., Rädle R., Rolland C., Gustafson L. SAM 2: Segment anything in images and videos. Proceedings of the International Conference on Learning Representations.

[44] Wang H., Qiao L., Jie Z., Huang Z., Feng C., Zheng Q., Liang X. X-SAM: From Segment Anything to Any Segmentation. Proceedings of the AAAI Conference on Artificial Intelligence, Singapore.

[45] Gu A., Dao T. (2024). Mamba: Linear-time sequence modeling with selective state spaces. arXiv.

[46] Wang Z., Zheng J.Q., Zhang Y., Cui G., Li L. (2024). Mamba-UNet: UNet-like pure visual Mamba for medical image segmentation. arXiv.

[47] Ma J., Li F., Wang B. (2024). U-Mamba: Enhancing long-range dependency for biomedical image segmentation. arXiv.

[48] Shi Z., Zhou H., Li L., Peng S., Liu R., Wan F., Zhu J., Zhang R., Chen J., Li L. (2026). LUNet: Language-infused UNet for precise *Camellia oleifera* pests and diseases segmentation in complex agricultural environment. Artif. Intell. Agric..

[49] Zheng S., Zhou H., Shi Z., Su F., Shi W., Liu R., Li L., Wan F. (2026). TB-DLossNet: Fine-grained segmentation of tea leaf diseases based on semantic-visual fusion. Plants.

[50] Zhou H., Li L., Peng S., Xu S., Shi Z., Xie B., Peng Y., Zhao B. (2026). RLEM-Net: Reinforcement learning enhanced multimodal segmentation for *Camellia oleifera* diseases based on semantic and visual features. Expert Syst. Appl..

[51] Jiang L., Dai B., Wu W., Loy C.C. Focal frequency loss for image reconstruction and synthesis. Proceedings of the IEEE/CVF International Conference on Computer Vision.

[52] Wang C., Zhang Y., Cui M., Ren P., Yang Y., Xie X., Hua X.S., Bao H., Xu W. Active boundary loss for semantic segmentation. Proceedings of the AAAI Conference on Artificial Intelligence.

[53] Zeng N., Li H., Wang Z., Liu W., Liu S., Alsaadi F.E., Liu X. (2021). Deep-reinforcement-learning-based images segmentation for quantitative analysis of gold immunochromatographic strip. Neurocomputing.

[54] Huang J., Xu Z., Zhou J., Liu T., Xiao Y., Ou M., Ji B., Li X., Yuan K. SAM-R1: Leveraging SAM for reward feedback in multimodal segmentation via reinforcement learning. Proceedings of the 39th Conference on Neural Information Processing Systems.

[55] Chen C., Xu J., Liao W., Ding H., Zhang Z., Yu Y., Zhao R. Focus-Then-Decide: Segmentation-assisted reinforcement learning. Proceedings of the AAAI Conference on Artificial Intelligence.

[56] Chen L.C., Zhu Y., Papandreou G., Schroff F., Adam H. Encoder-decoder with atrous separable convolution for semantic image segmentation. Proceedings of the European Conference on Computer Vision.

[57] Ronneberger O., Fischer P., Brox T. U-Net: Convolutional networks for biomedical image segmentation. Proceedings of the Medical Image Computing and Computer-Assisted Intervention.

[58] Jain J., Li J., Chiu M.T., Hassani A., Orlov N., Shi H. OneFormer: One Transformer to rule universal image segmentation. Proceedings of the IEEE/CVF Conference on Computer Vision and Pattern Recognition.

[59] Zhao H., Shi J., Qi X., Wang X., Jia J. Pyramid scene parsing network. Proceedings of the IEEE Conference on Computer Vision and Pattern Recognition.

[60] Wang J., Sun K., Cheng T., Jiang B., Deng C., Zhao Y., Liu D., Mu Y., Tan M., Wang X. (2021). Deep high-resolution representation learning for visual recognition. IEEE Trans. Pattern Anal. Mach. Intell..

[61] Cheng B., Misra I., Schwing A.G., Kirillov A., Girdhar R. Masked-attention mask transformer for universal image segmentation. Proceedings of the IEEE/CVF Conference on Computer Vision and Pattern Recognition.

[62] Xing Z., Ye T., Yang Y., Liu G., Zhu L. (2024). SegMamba: Long-range Sequential Modeling Mamba For 3D Medical Image Segmentation. arXiv.

[63] Wu R., Liu Y., Liang P., Chang Q. (2025). H-vmunet: High-order Vision Mamba UNet for medical image segmentation. Neurocomputing.

[64] Ma J., He Y., Li F., Han L., You C., Wang B. (2024). Segment anything in medical images. Nat. Commun..

[65] Feng J., Chao X. (2022). Apple Tree Leaf Disease Segmentation Dataset, V1.

